# Lcn2 Promotes Ferroptosis in Intracerebral Hemorrhage by Targeting Keap1 Tyr572 and Restricting Nrf2 Activation

**DOI:** 10.1002/mco2.70920

**Published:** 2026-08-16

**Authors:** Ya‐nan Dou, Hongkang Hu, Qinghua Li, Guangming Wang, Lijun Hou, Junbin Liu, Buyi Zheng, Ying Li, Guohan Hu, Zhou Fei, Xia Li, Li Wang, Jialiang Wei, Lei Jiang, Xiaowei Fei

**Affiliations:** ^1^ Department of Neurosurgery Xijing Hospital Air Force Military Medical University Xi'an Shaanxi China; ^2^ Department of Neurosurgery Changzheng Hospital Naval Medical University Shanghai China; ^3^ Department of Pediatrics Shanghai Ninth People's Hospital Shanghai JiaoTong University School of Medicine Shanghai China; ^4^ Department of Orthopedics Changzheng Hospital Naval Medical University Shanghai China; ^5^ Department of Neurosurgery Wenzhou People's Hospital Wenzhou Zhejiang China; ^6^ Department of Pathology Changzheng Hospital Naval Medical University Shanghai China; ^7^ Neurovascular Center Changhai Hospital Naval Medical University Shanghai China; ^8^ Department of Neurosurgery Zhongshan Hospital Fudan University Shanghai China

**Keywords:** intracerebral hemorrhage, lipocalin‐2, ferroptosis, nuclear factor erythroid 2‐related factor 2, oxidative stress

## Abstract

Ferroptosis is a major driver of neuronal injury after intracerebral hemorrhage (ICH). Lipocalin‑2 (Lcn2), an iron‑binding transport protein, modulates neuronal iron homeostasis and has been reported to exert both neurotoxic and neuroprotective actions. The precise contribution of Lcn2 to ferroptosis following ICH remains unresolved. Here, it is found that Lcn2 concentrations were significantly higher in hematoma than in arterial blood from the same patients. Proteomics and inflammation‑array data converged on ferroptosis and the Nrf2‐Keap1 axis as key pathways altered in Lcn2^fl/fl^Nestin^Cre^ mice. Lcn2 deletion improved mitochondrial integrity and reduced lipid peroxidation in primary neurons. Mechanistically, Lcn2 competitively bound Keap1 at Tyr572, restricting Nrf2 nuclear translocation and thereby promoting ferroptosis. Knockdown of Nrf2 or pharmacological inhibition with HY‑149508 (Nrf2 inhibitor) abrogated the Lcn2‑knockout‐mediated rescue of neuronal activity and mitochondrial function. Notably, treatment with a Keap1 Tyr572Ala mutant by adeno‐associated viruses conferred robust neuroprotection only in the presence of Lcn2. Lcn2 promotes ferroptosis neuronal death after ICH by engaging Keap1 at Tyr572 to suppress Nrf2 activation. The therapeutic efficacy of Keap1(Tyr572Ala) is contingent on Lcn2 expression, revealing an unexpected context dependence within the Lcn2‐Nrf2 signaling axis. These findings identify a tractable molecular interface for precision modulation of ferroptosis in ICH.

## Graphical abstract

This study reveals that Lcn2 regulates ferroptosis after ICH via the Keap1/Nrf2 axis. Neuronal Lcn2 deletion reduces lipid peroxidation, restores mitochondrial function, and improves ferroptosis. Keap1 Tyr572Ala mutant acts as a molecular switch whose effects invert with Lcn2 status. These findings refine current models of redox control in hemorrhagic brain injury and suggest biomarker‑guided strategies for precise modulation of ferroptosis.

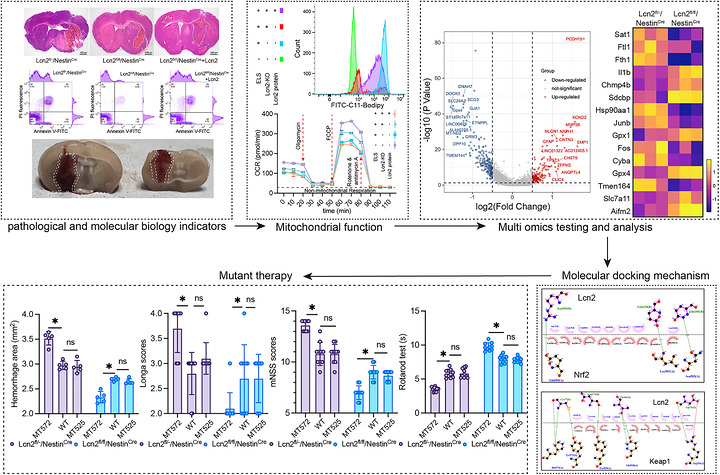

## 1 Introduction

Stroke, including both ischemic stroke and hemorrhagic stroke, ranks as the world's second leading cause of death. Intracerebral hemorrhage (ICH) is among the deadliest forms of stroke, carrying a case‑fatality rate exceeding 50% [1]. Despite growing interest and multiple clinical trials, current management remains largely supportive and truly effective disease‑modifying therapies are scarce [2]. Secondary injury after ICH is driven in part by hemolysis and hemoglobin degradation, which liberate large quantities of iron. Iron accumulation fuels lipid peroxidation, reactive oxygen species (ROS) generation, mitochondrial dysfunction, and neuroinflammation‐hallmarks that converge on ferroptosis, an iron‑dependent form of regulated cell death [3]. In experimental ICH, brain iron content can triple and remain elevated for at least 1 month [4]. Strategies that restore cerebral iron homeostasis and thereby dampen ferroptosis and neuroinflammation represent promising therapeutic avenues [5].

Lipocalin‑2 (Lcn2), the second member of the lipocalin family, is an iron‑trafficking and inflammatory mediator with established roles in lipid transport, neuroinflammation, and ferritin handling [6, 7]. Clinically, serum Lcn2 is markedly elevated in ICH and correlates positively with C‑reactive protein, glycemia, NIH Stroke Scale score and hematoma volume, while inversely correlating with Glasgow Coma Scale score‐implicating high Lcn2 as a risk factor for poor outcome [8]. In mouse ICH models, Lcn2 is robustly upregulated in microglia and astrocytes and contributes to microglial ferroptosis [9]. Mechanistic work suggests that Lcn2 selectively suppresses the ferritin light chain in microglia without affecting the heavy chain, linking ferritin dysregulation to ferroptosis and subsequent neuronal injury [10]. In patients with subarachnoid hemorrhage (SAH), cerebrospinal fluid Lcn2 is increased and predicts poor 3‑month outcomes [11]. Lcn2 has also been associated with white‑matter hyperintensity and blood–brain barrier (BBB) disruption after SAH‐findings mitigated in Lcn2‑deficient mice [12] and may promote ultra‑early thrombosis in experimental SAH [13].

Notably, Lcn2 can play context‑dependent, even opposing, roles in the nervous system. In acute ischemic stroke (AIS), increased Lcn2 has been reported to support synaptic plasticity and augment the anti‑inflammatory cytokine IL‑10, alleviating neuroinflammation [14]. Lcn2 can also strengthen BBB integrity by upregulating tight‑junction proteins in TNF‑α‑induced injury models [15]. In addition, there are many studies reporting the regulation of Lcn2 on the BBB integrity under various pathological conditions. However, most of them reported a promoting role of Lcn2 on the BBB disruption and neuroinflammation [16, 17]. Such bidirectionality may reflect distinct astrocyte responses: pro‑inflammatory A1 astrocytes activate NF‑κB, elevate Lcn2, and secrete interferon‑γ, whereas A2 astrocytes engage JAK/STAT3, suppress Lcn2, and release trophic factors such as BDNF and VEGF [18, 19]. These observations underscore that Lcn2 may simultaneously drive neurotoxicity and neuroprotection, depending on cellular context and signaling state. The molecular mechanism underlying Lcn2's context‐dependent switch between neurotoxicity and neuroprotection in ICH remains largely unknown, which this study aims to address.

Here, we interrogate the role of neuronal Lcn2 in ferroptosis after ICH. We employ neuron‑specific Lcn2 knockout mice (Lcn2^fl/−^Nestin^Cre^ and Lcn2^fl/fl^Nestin^Cre^) and primary neurons, combined with lentiviral and adeno‑associated viral delivery and CRISPR/Cas9 editing, to manipulate ferroptosis regulators, including nuclear factor erythroid 2‐related factor 2 (Nrf2) and its cytoplasmic repressor Kelch‑like ECH‑associated protein 1 (Keap1). Using biochemical and molecular assays, we quantify brain edema, mitochondrial function, oxidative stress, apoptosis, and inflammatory mediators. These studies aim to map the regulatory interface between Lcn2 and the Nrf2/Keap1 axis, identify actionable binding sites, and define how modulation of this pathway controls ferroptosis and neurological outcomes after ICH.

## 2 Results

### 2.1 LCN2 Is Markedly Enriched in Hematoma Compared With Arterial Blood From Patients With ICH

**FIGURE 1:**
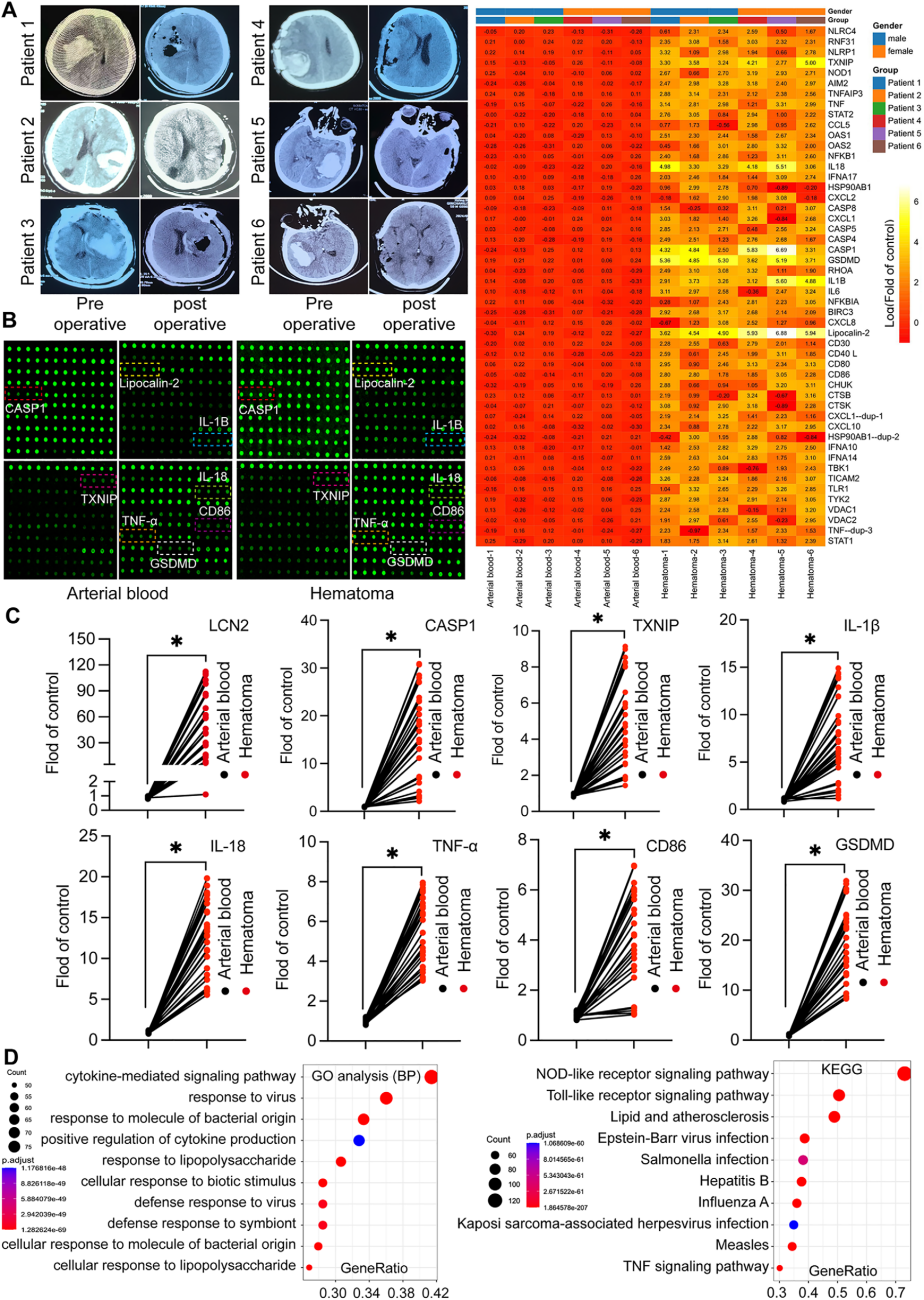
Lcn2 is present at high levels in hematomas of ICH patients. (A) CT results of six patients before and after surgery. (B) Representative output from the 200‐plex protein array (left). Heatmap of the top 50 differentially expressed genes from the protein microarray (rigght). (C) The levels of LCN2 (*t* = 7.874, df = 29), CASP1 (*t* = 9.029, df = 29), TXNIP (*t* = 8.998, df = 29), IL‐1β (*t* = 8.477, df = 29), IL‐18 (*t* = 15.620, df = 29), TNF‐α (*t* = 15.59, df = 29), CD86 (*t* = 9.870, df = 29), and GSDMD (*t* = 14.450, df = 29) in hematoma and arterial blood from 30 patients were measured by ELISA. (D) GO and KEGG analysis of differentially expressed genes from protein microarrays. The data (C) were analyzed using paired *t* test. *p < 0.05 represents a statistically significant difference between the two groups. ns: no statistical difference.

With informed consent, we collected preoperative arterial blood and intraoperatively removed hematoma from six patients with ICH. Postoperative emergency CT confirmed hematoma evacuation rates >95% in all cases (Figure 1A). A protein array profiling 200 targets—encompassing inflammatory cytokines, chemokines, and apoptosis‑related factors—revealed substantially higher levels of LCN2, CASP1, TXNIP, IL‑1β, IL‑18, TNF‑α, CD86, and GSDMD in hematoma than in paired arterial blood. LCN2 exhibited the largest difference, increasing up to 6.88‑fold (Figure 1B). This pattern was independently validated in an expanded cohort of 30 patients (Figure 1C). Gene Ontology and KEGG enrichment of differentially expressed factors highlighted canonical innate immune pathways, including NOD‑like and Toll‑like receptor signaling (Figure 1D). In addition, the ratio of LCN2 at the hemorrhage site to that in arterial blood is correlated with patients’ baseline clinical characteristics, bleeding volume, and CS score, but is not associated with the hemorrhage location (Table S1). Together, these data implicate high intralesional LCN2 as a putative driver of peri‑hematomal neurotoxicity in ICH.

### 2.2 Neuronal Deletion of Lcn2 Ameliorates ICH Outcomes, Whereas Exogenous Lcn2 Worsens Disease

**FIGURE 2:**
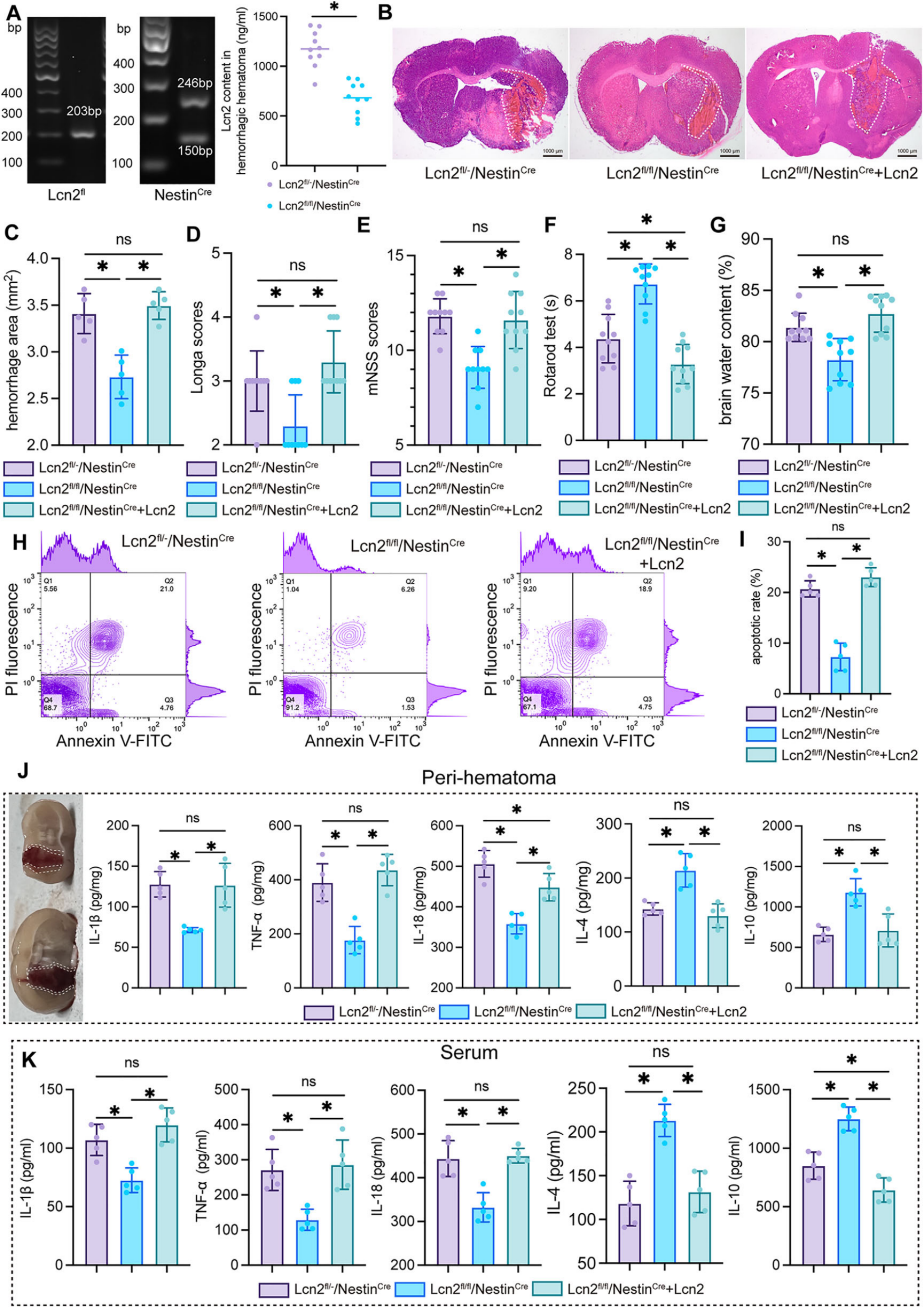
Lcn2^fl/fl^Nestin^Cre^ mice have a better prognosis after ICH compared to Lcn2^fl/−^Nestin^Cre^ mice. (A) Agarose gel electrophoresis for gene identification in transgenic mice. Lcn2^fl/fl^ is homozygous with the Loxp site inserted. Lcn2^fl/−^ is an allele lacking loxP locus. ELISA was used to assess the effect of Lcn2 knockout on Lcn2 expression at the hemorrhage site. *t* = 6.359, df = 18. (B) HE staining was performed on the largest cross‐section of the bleeding area for each group of mice. (C) Comparison of hemorrhagic areas in each group of mice. *F* (2, 12) = 21.56, *p* = 0.0001. (D) Longa scores in each group of mice. *F* (2, 27) = 11.47, *p* = 0.0002. (E) mNSS scores in each group of mice. *F* (2, 27) = 15.71, *p* < 0.0001. (F) The rotarod test measures the duration that mice can maintain their balance on a rotating rod. *F* (2, 27) = 36.70, *p* < 0.0001. (G) brain water content in each group of mice. *F* (2, 27) = 16.94, *p* < 0.0001. (H) Assess the level of apoptosis in the brain tissue surrounding the hemorrhage in each group of mice using flow cytometry. (I) Quantification of results in Panel H. *F* (2, 12) = 80.67, *p* < 0.0001. (J) The Elisa test detected the expression levels of inflammation‐related factors in the brain tissue surrounding the hemorrhage. IL 1β: *F* (2, 12) = 15.99, *p* = 0.0004. TNF‐α: *F* (2, 12) = 26.74, *p* < 0.0001. IL‐18: *F* (2, 12) = 29.30, *p* < 0.0001. IL‐4: *F* (2, 12) = 19.59, *p =* 0.0002. IL‐10: *F* (2, 12) = 41.44, *p* < 0.0001. (K) The Elisa test detected the expression levels of inflammation‐related factors in mice serum. IL 1β: *F* (2, 12) = 17.97, *p* = 0.0002. TNF‐α: *F* (2, 12) = 12.17, *p* = 0.0013. IL‐18: *F* (2, 12) = 21.35, *p* < 0.0001. IL‐4: *F* (2, 12) = 25.67, *p* < 0.0001. IL‐10: *F* (2, 12) = 15.93, *p* = 0.0004. The data were analyzed using Student's *t* test (A) or one‐way analysis of variance (C–G, I–K) and all data are expressed as the mean ± standard deviation. *p < 0.05 represents a statistically significant difference between the two groups. ns: no statistical difference.

As previously published by our research group [10, 20, 21, 22], to define the neuronal contribution of Lcn2 to ICH, we generated neuron‑specific conditional knockout mice (Lcn2^fl/fl^Nestin^Cre^) and confirmed homozygosity of the LoxP sites (203 bp) and demonstrated that, in the presence of Cre recombinase (246 bp), neuronal Lcn2 knockout can effectively reduce Lcn2 levels in the hematoma. (Figure 2A). In brief, baseline characterization of the conditional knockout mice showed no significant differences compared with wild‐type mice in the absence of ICH, and confirmed efficient and neuron‐specific deletion of Lcn2. In addition, using Lcn2^fl/–^ mice as controls preserves normal Lcn2 expression while excluding potential nonspecific effects of LoxP sites on the experimental outcomes [10, 20, 21, 22].

Relative to littermate controls, Lcn2^fl/fl^Nestin^Cre^ mice exhibited smaller hematoma volumes (Figure 2B–C), lower Longa and mNSS scores (Figure 2D–E), longer latency to fall on the rotarod (Figure 2F), and reduced brain edema (Figure 2G) at Day 3 after ICH induction. Flow cytometry further showed decreased apoptosis in peri‑hematomal tissue of Lcn2^fl/fl^Nestin^Cre^ mice compared with Lcn2^fl/−^Nestin^Cre^ controls (Figure 2H–I). ELISAs of brain tissue and serum demonstrated reduced pro‑inflammatory mediators (IL‑1β, TNF‑α, IL‑18) and increased anti‑inflammatory cytokines (IL‑4, IL‑10) in the knockout (Figure 2J–K). Conversely, in situ administration of recombinant Lcn2 into Lcn2^fl/fl^Nestin^Cre^ mice after ICH negated these benefits, exacerbating injury. These findings indicate that neuronal Lcn2 aggravates ICH pathology.

### 2.3 Multi‑Omics Implicate Ferroptosis and the Nrf2‐Keap1 Axis in Lcn2‑Mediated Injury

**FIGURE 3:**
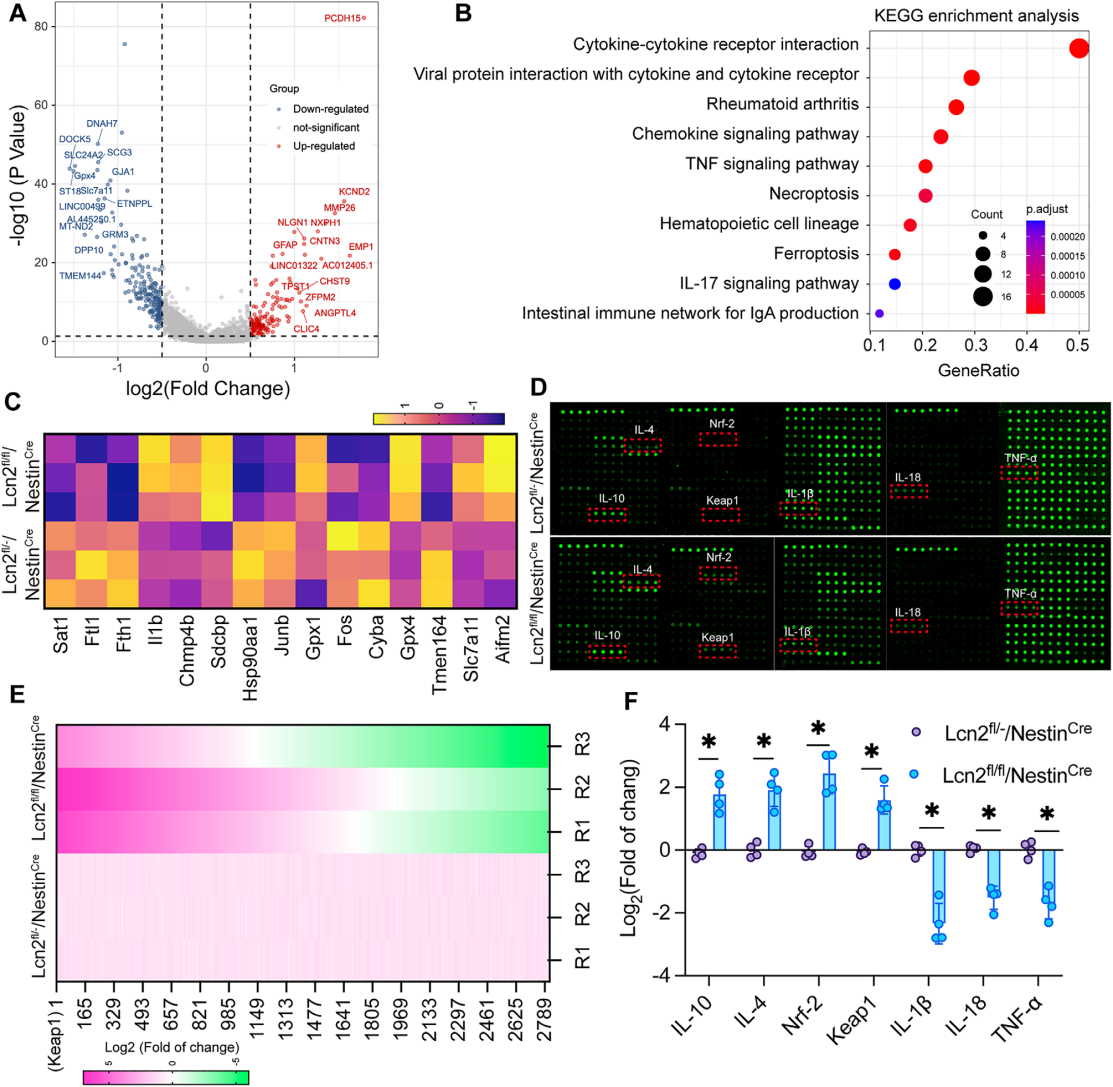
Proteomics and inflammation chip suggest that Lcn2 knockout is related to ferroptosis and Nrf2/Keap1. (A) Volcano plot of differential genes in Lcn2^fl/fl^Nestin^Cre^ mice compared to Lcn2^fl/−^Nestin^Cre^ mice. (B) Downregulation of differential gene KEGG enrichment analysis. (C) Expression levels of ferroptosis‐related gene sets. (D) Representative Glass Slide images of the 2808 inflammation‐related protein markers detected in the brain tissue surrounding the hemorrhage. (E) Inflammation chip indicator heatmap. (F) Quantification of representative indicators for chips. IL‐10: *t* = 6.474, df = 6, *p* = 0.0006. IL‐4: *t* = 6.727, df = 6, *p* = 0.0005. Nrf‐2: *t* = 7.309, df = 6, *p* = 0.0003. Keap1: *t* = 7.233, df = 6, *p* = 0.0004. IL‐1β: *t* = 6.966, df = 6, *p* = 0.0004. IL‐18: *t* = 8.110, df = 6, *p* = 0.0002. TNF‐α: *t* = 6.390, df = 6, *p =* 0.0007. The data were analyzed using Student's *t*‐test and all data are expressed as the mean ± standard deviation. *p < 0.05 represents a statistically significant difference between the two groups. ns: no statistical difference.

Proteomic profiling of peri‑hematomal tissue revealed that control mice, relative to neuronal Lcn2 knockouts, had reduced abundance of ferroptosis defense proteins GPX4 and SLC7A11, and increased GFAP, EMP26 and KCND2 (Figure 3A). Enrichment analyses of downregulated proteins pointed to TNF‑α, IL‑17, and ferroptosis pathways (Figure 3B; Figure S1A–C). Among 15 curated ferroptosis‑related genes, including Stat1 and Ftl1, expression was broadly remodeled by Lcn2 deletion (Figure 3C). A high‑content inflammatory array (2808 analytes) further showed that Lcn2 knockout shifted the cytokine milieu toward an anti‑inflammatory state (elevated IL‑4 and IL‑10) and identified Keap1—the canonical inhibitor of Nrf2—as one of the most increased proteins (Figure 3D,E). Aggregate quantification indicated upregulation of both Nrf2 and Keap1 in the knockout tissue (Figure 3F), suggesting engagement of the Nrf2/Keap1 antioxidant pathway in the ferroptosis attenuation associated with Lcn2 loss.

### 2.4 Neuronal Lcn2 Deletion Suppresses Ferroptosis in Vivo After ICH

Consistent with the omics data, peri‑hematomal tissue from Lcn2^fl/fl^Nestin^Cre^ mice showed increased transcriptional and protein levels of the ferroptosis‑suppressive antioxidant machinery GPX4 and xCT/SLC7A11 (Figure S2A–C), together with altered iron‑handling proteins ferroportin and hepcidin (Figure S3A,B). Immunofluorescence confirmed neuronal upregulation of GPX4 and xCT (Figure S2D). Histochemical iron staining and biochemical assays demonstrated reduced iron deposition in brain tissue (Figure S2E,F) and serum (Figure S2G) of knockout mice. Markers of lipid peroxidation were diminished (MDA, 4‑HNE; Figure S2H,I), whereas antioxidant capacity was elevated (GSH and SOD; Figure S2J–K). These results support a reduction in ferroptotic stress when neuronal Lcn2 is ablated.

### 2.5 In Vitro Modeling Confirms That Lcn2 Loss Preserves Mitochondrial Function and Limits Lipid Peroxidation

**FIGURE 4:**
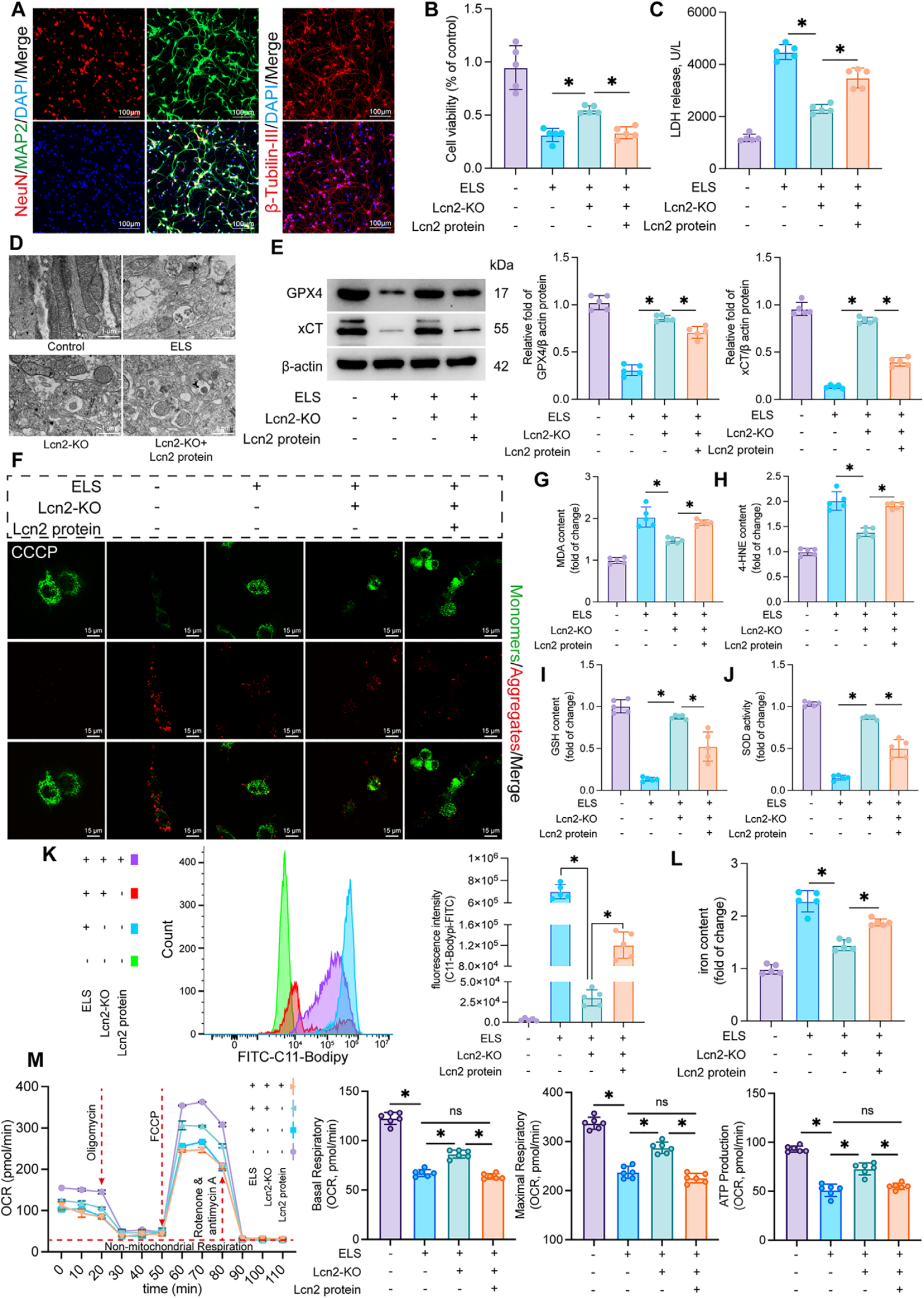
Lcn2‐KO improves mitochondrial function and lipid peroxidation in primary neurons, inhibiting ferroptosis. (A) The primary neurons were identified using the markers NeuN, MAP2, and β‐tubulin III. (B) The CCK‐8 assay is used for detecting cell viability. *F* (3, 16) = 33.97, *p* < 0.0001. C LDH release level detection. *F* (3, 16) = 147.9, *p* < 0.0001. (D) TEM is used to detect the morphology of cell mitochondria. (E) Expression levels of GPX4 and xCT protein. GPX4: *F* (3, 16) = 135.6, *p* < 0.0001. xCT: *F* (3, 16) = 370.9, *p* < 0.0001. (F) JC‐1 is used to detect mitochondrial membrane potential levels, with CCCP‐treated cells serving as a positive control. (G) Detection of MDA levels. *F* (3, 16) = 61.23, *p* < 0.0001. (H) Detection of 4‐HNE levels. *F* (3, 16) = 90.80, *p <* 0.0001. (I) Detection of GSH levels. *F* (3, 16) = 81.43, *p <* 0.0001. (J) Detection of SOD activity. *F* (3, 16) = 232.90, *p* < 0.0001. (K) C‐11 Bodipy flow cytometry is used to assess lipid peroxidation levels. *F* (3, 16) = 452.6, *p* < 0.0001. (L) Detection of cellular iron ion content. *F* (3, 16) = 97.05, *p* < 0.0001. (M) The Seahorse experiment is used to detect mitochondrial function, data are expressed as the mean ± standard deviation. Basal respiratory: *F* (3, 20) = 238.6, *p* < 0.0001. Maximal respiratory: *F* (3, 20) = 119.4, *p* < 0.0001. ATP production: *F* (3, 20) = 92.96, *p* < 0.0001. The data were analyzed using one‐way analysis of variance and all data are expressed as the mean ± standard deviation. *p < 0.05 represents a statistically significant difference between the two groups. ns: no statistical difference.

To recapitulate ICH‑like stress in vitro, primary neurons—validated by NeuN, MAP2, and β‑tubulin III staining (Figure 4A)—were exposed to erythrocyte lysis solution (ELS). CRISPR/Cas9‑mediated Lcn2 knockout was established (Figure S4A–C). The CCK experiment is used to detect the effects of different concentrations of Lcn2 protein treatment on cell viability, and 140 ng/mL recombinant Lcn2 was identified as the maximal non‑toxic dose (Figure S5). Under ELS, Lcn2‑KO enhanced cell viability (Figure 4B) and reduced LDH release (Figure 4C). Ultrastructurally, mitochondria exhibited improved morphology, an effect reversed by exogenous Lcn2 (Figure 4D). Lcn2‑KO increased GPX4 and xCT at mRNA and protein levels (Figure 4E; Figure S6A–B) and modulated iron‑handling proteins Ferroportin and Hepcidin (Figure S7A,B). Mitochondrial membrane potential, reduced by ELS, was restored by Lcn2‑KO (Figure 4F). Lipid peroxidation (MDA, 4‑HNE) decreased (Figure 4G,H), while GSH and SOD were elevated (Figure 4I,J). The ferroptosis inhibitor Ferrostain‑1 mimicked, and the ferroptosis agonist RSL3 counteracted, these protective effects. Notably, Lcn2‑KO mitigated RSL3‑induced lipid peroxidation (Figure 4K; Figures S8 and S9). Both RhoNox‑1 staining and biochemical assays showed reduced neuronal iron with Lcn2‑KO (Figure 4L; Figure S10A,B). Seahorse analyses demonstrated improved oxidative phosphorylation‐higher OCR, basal and maximal respiration, and ATP production‐in Lcn2‑KO neurons (Figure 4M). Thus, loss of Lcn2 restrains ferroptosis and preserves mitochondrial bioenergetics in vitro.

### 2.6 Lcn2 Deletion Promotes Nrf2 Nuclear Translocation and Reprograms Ferroptosis‑Related Gene Expression

**FIGURE 5:**
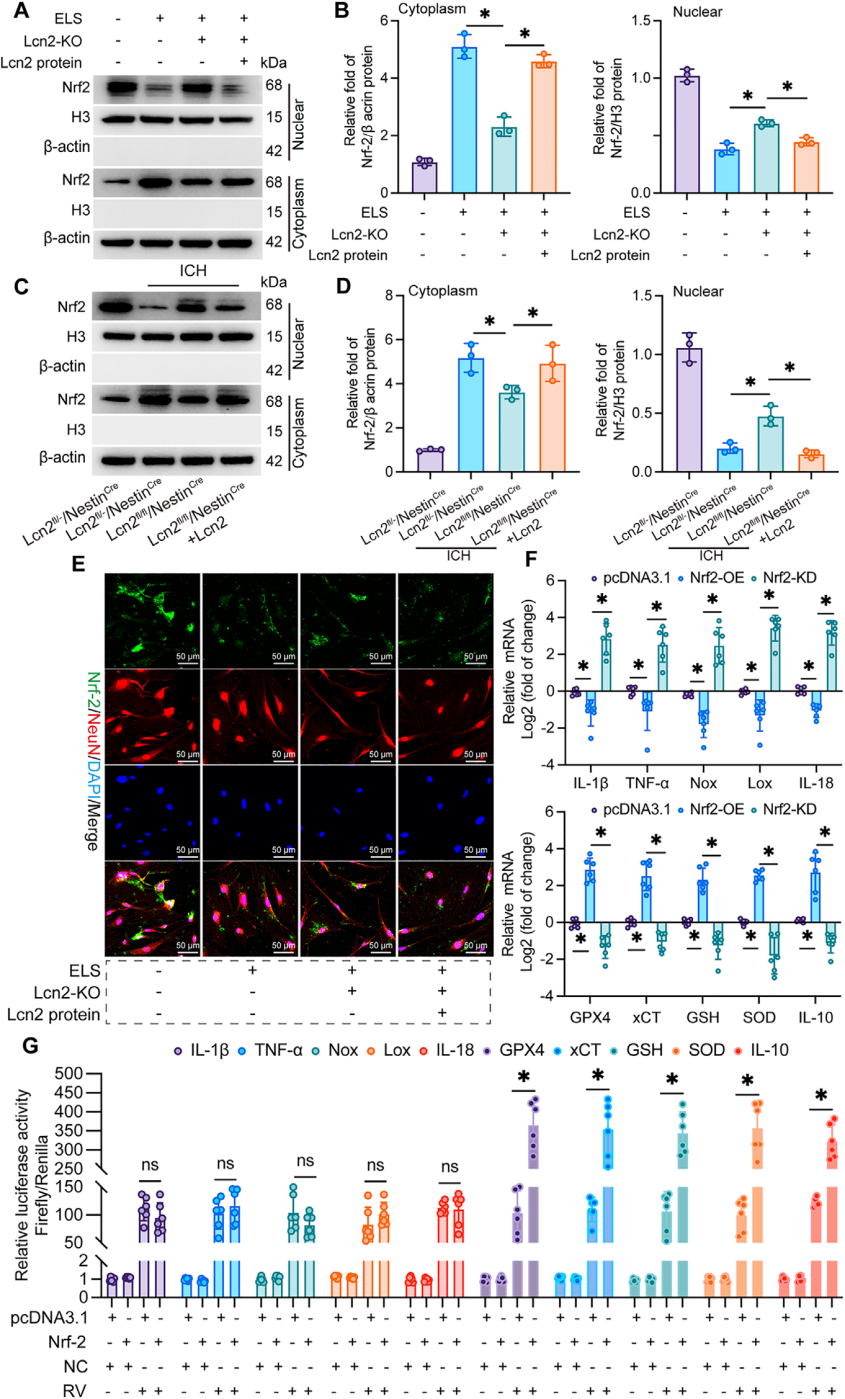
Knockout of Lcn2 in both in vivo and in vitro experiments promoted the nuclear translocation of Nrf2. (A) Nrf2 nuclear translocation levels in each group in cell experiments. (B) Quantification of results in Panel A. Nrf2/β actin: *F* (3, 8) = 123.4, *p* < 0.0001. Nrf2/H3: *F* (3, 8) = 130.00, *p* < 0.0001. (C) Nrf2 nuclear translocation levels in each group in mice experiments. (D) Quantification of results in Panel C. Nrf2/β actin: *F* (3, 8) = 36.76, *p* < 0.0001. Nrf2/H3: *F* (3, 8) = 83.23, *p <* 0.0001. (E) Co‐localization levels of Nrf2 and NeuN. F The effect of intervening in Nrf2 expression on the mRNA levels of inflammation and ferroptosis‐related proteins. IL‐1β: *F* (2, 15) = 61.52, *p <* 0.0001. TNF‐α: *F* (2, 15) = 30.55, *p <* 0.0001. Nox: *F* (2, 15) = 55.85, *p* < 0.0001. Lox: *F* (2, 15) = 88.51, *p* < 0.0001. IL‐18: *F* (2, 15) = 141.8, *p* < 0.0001. GPX4: *F* (2, 15) = 99.89, *p* < 0.0001. xCT: *F* (2, 15) = 65.07, *p <* 0.0001. GSH: *F* (2, 15) = 60.58, *p <* 0.0001. SOD: *F* (2, 15) = 80.06, *p <* 0.0001. IL‐10: *F* (2, 15) = 48.94, *p <* 0.0001. (G) The dual luciferase reporter assay is used to detect the transcriptional regulatory effects of Nrf2 on genes related to inflammation and ferroptosis. IL‐1β: *F* (3, 20) = 72.60, *p <* 0.0001. TNF‐α: *F* (3, 20) = 54.92, *p* < 0.0001. Nox: *F* (3, 20) = 48.85, *p* < 0.0001. Lox: *F* (3, 20) = 48.71, *p* < 0.0001. IL‐18: *F* (3, 20) = 101.9, *p* < 0.0001. GPX4: *F* (3, 20) = 128.2, *p* < 0.0001. xCT: *F* (3, 20) = 119.1, *p* < 0.0001. GSH: *F* (3, 20) = 146.9, *p <* 0.0001. SOD: *F* (3, 20) = 129.7, *p <* 0.0001. IL‐10: *F* (3, 20) = 264.8, *p <* 0.0001. The data were analyzed using one‐way analysis of variance and all data are expressed as the mean ± standard deviation. *p < 0.05 represents a statistically significant difference between the two groups. ns: no statistical difference.

Subcellular fractionation and immunoblotting showed increased nuclear Nrf2 in both brain tissue and primary neurons lacking Lcn2 (Figure 5A–D), corroborated by immunofluorescence (Figure 5E). In primary neurons, Nrf2 overexpression elevated GPX4, xCT, GSH, SOD, and IL‑10 and reduced IL‑1β, TNF‑α, NOX, LOX, and IL‑18 (Figure 5F). Dual luciferase assay showed that Nrf2 directly transactivates GPX4, xCT, GSH, SOD, and IL‐10 promoters, but has no significant direct effect on transcriptional activation of pro‐inflammatory genes IL‑1β, TNF‑α, NOX, LOX, and IL‑18. This suggests that Nrf2's inhibition of these factors may occur indirectly (Figure 5G). In vivo, Nrf2 knockdown worsened ICH outcomes—larger hemorrhage, poorer Longa and mNSS scores, shorter rotarod times, increased edema, and apoptosis—and skewed cytokines toward a pro‑inflammatory profile (Figure S10A–I), underscoring Nrf2's protective role.

### 2.7 Nrf2 Is Required for the Lcn2‑KO‑Mediated Rescue of Neuronal Function

**FIGURE 6:**
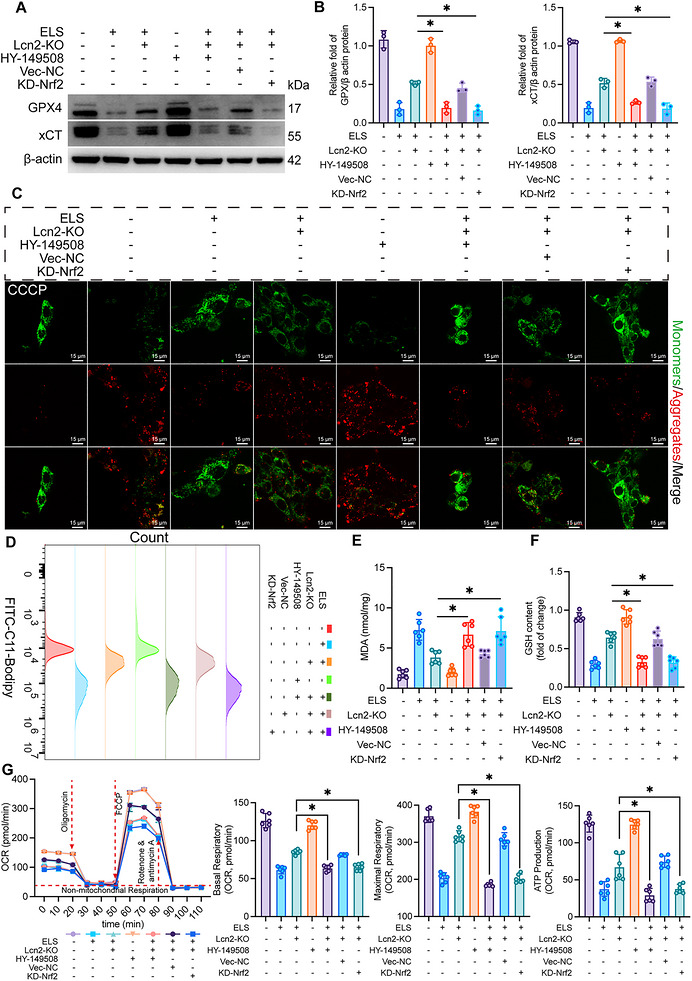
HY‐149508 and Nrf2 knockdown inhibit Lcn2‐induced mitochondrial function recovery and lipid peroxidation. (A) Expression levels of GPX4 and xCT protein. (B) Quantification of results in Panel A. GPX4: *F* (6, 14) = 79.28, *p <* 0.0001. xCT: *F* (6, 14) = 172.20, *p* < 0.0001. (C) JC‐1 is used to detect mitochondrial membrane potential levels, with CCCP‐treated cells serving as a positive control. (D) C‐11 Bodipy flow cytometry is used to assess lipid peroxidation levels. (E) Detection of MDA levels. *F* (6, 35) = 28.31, *p <* 0.0001. (F) Detection of GSH levels. *F* (6, 35) = 76.75, *p <* 0.0001. (G) The Seahorse experiment is used to detect mitochondrial function, data are expressed as the mean ± standard deviation. Basal respiratory: *F* (6, 35) = 160.8, *p <* 0.0001. Maximal respiratory: *F* (6, 35) = 200.80, *p <* 0.0001. ATP Production: *F* (6, 35) = 93.32, *p* < 0.0001. The data were analyzed using one‐way analysis of variance and all data are expressed as the mean ± standard deviation. *p < 0.05 represents a statistically significant difference between the two groups. ns: no statistical difference.

In cultured neurons, Nrf2 knockdown or pharmacological inhibition with HY‑149508 (10 µM, 24 h) alone had minimal effects under basal conditions but, under ELS, abolished the improvements in viability and LDH release conferred by Lcn2‑KO (Figure S12A,B). Nrf2 interference downregulated GPX4 and xCT (Figure 6A,B; Figure S12C), decreased mitochondrial membrane potential (Figure 6C), and increased mitochondrial and total ROS as well as cellular iron (Figure S12D–F). Lipid peroxidation intensified (Figure 6D), and MDA and 4‑HNE rose while GSH and SOD fell (Figures 6E,F; Figure S12G,H). Seahorse analyses confirmed reduced OCR, basal/maximal respiration, and ATP production compared with Lcn2‑KO alone (Figure 6G). Thus, Nrf2 activity is necessary for the anti‑ferroptotic and bioenergetic benefits of Lcn2 loss.

### 2.8 Lcn2 Competitively Engages Keap1 at Tyr572 to Regulate Nrf2 Nuclear Translocation

**FIGURE 7:**
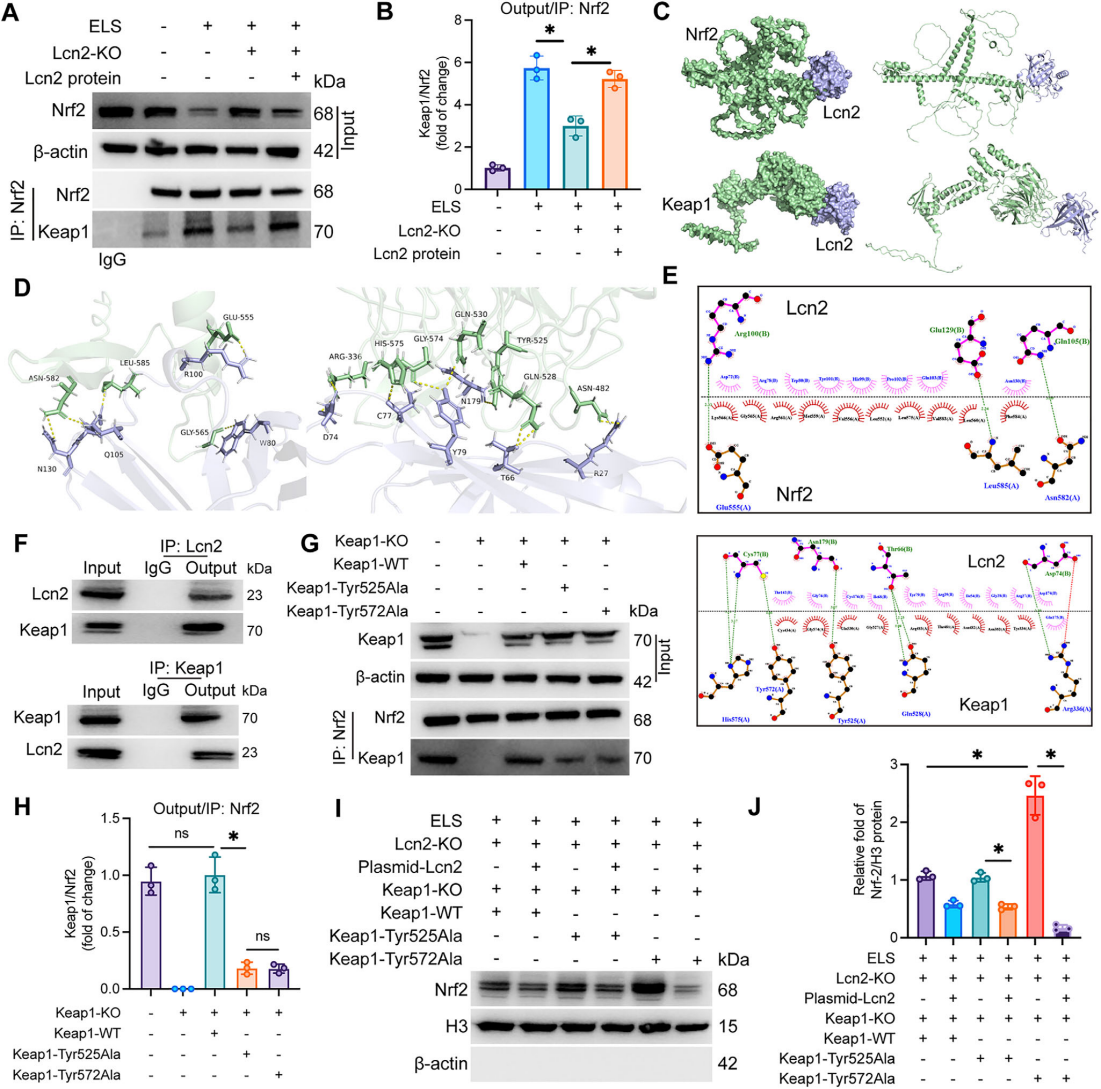
Lcn2 competitively binds to Keap1 at the Tyr572 site. (A) Co‐IP experiments were conducted to detect the binding ability of Nrf2 and Keap1 in each group. (B) Quantification of results in Panel A. *F* (3, 8) = 78.83, *p* < 0.0001. (C) Nrf2‐Lcn2 and Keap1‐Nrf2 predicted 3D binding models. (D) Nrf2‐Lcn2 and Keap1‐Nrf2 predict 3D binding sites. (E) Nrf2‐Lcn2 and Keap1‐Nrf2 predict 2D binding sites. (F) Co‐IP experiments were conducted to detect the binding ability of Lcn2 and Keap1. (G) Co‐IP detection of the effect of Keap1 mutants on Nrf2‐Keap1 binding. (H) Quantification of results in Panel G. *F* (4, 10) = 77.04, *p <* 0.0001. (I) WB is used to detect the effect of Keap1 mutants on the nuclear translocation of Nrf2. (J) Quantification of results in Panel I. *F* (5, 12) = 87.33, *p <* 0.0001. The data were analyzed using one‐way analysis of variance and all data are expressed as the mean ± standard deviation. *p < 0.05 represents a statistically significant difference between the two groups. ns: no statistical difference.

Keap1 restrains Nrf2 in the cytoplasm under homeostasis. Co‑immunoprecipitation demonstrated that Lcn2 deletion weakened Keap1‐Nrf2 binding, whereas recombinant Lcn2 restored it (Figure 7A,B). Molecular docking predicted Lcn2‐Keap1 interfaces at Tyr525 and Tyr572—sites also implicated in Nrf2–Keap1 interactions (Figure 7C–E)—and Co‑IP verified Lcn2‐Keap1 association (Figure 7F). Mutating Keap1 at Tyr525 or Tyr572 impaired Nrf2 binding (Figure 7G,H). Functionally, Keap1 Tyr572Ala promoted Nrf2 nuclear accumulation when Lcn2 was absent, but this effect was attenuated upon Lcn2 re‑expression (Figure 7I,J). These findings indicate that Lcn2 competes with Nrf2 for Keap1 at Tyr572, thereby limiting Nrf2 nuclear translocation.

### 2.9 Keap1 Tyr572 Mutation Differentially Modulates Neuronal Phenotypes Depending on Lcn2 Status

**FIGURE 8:**
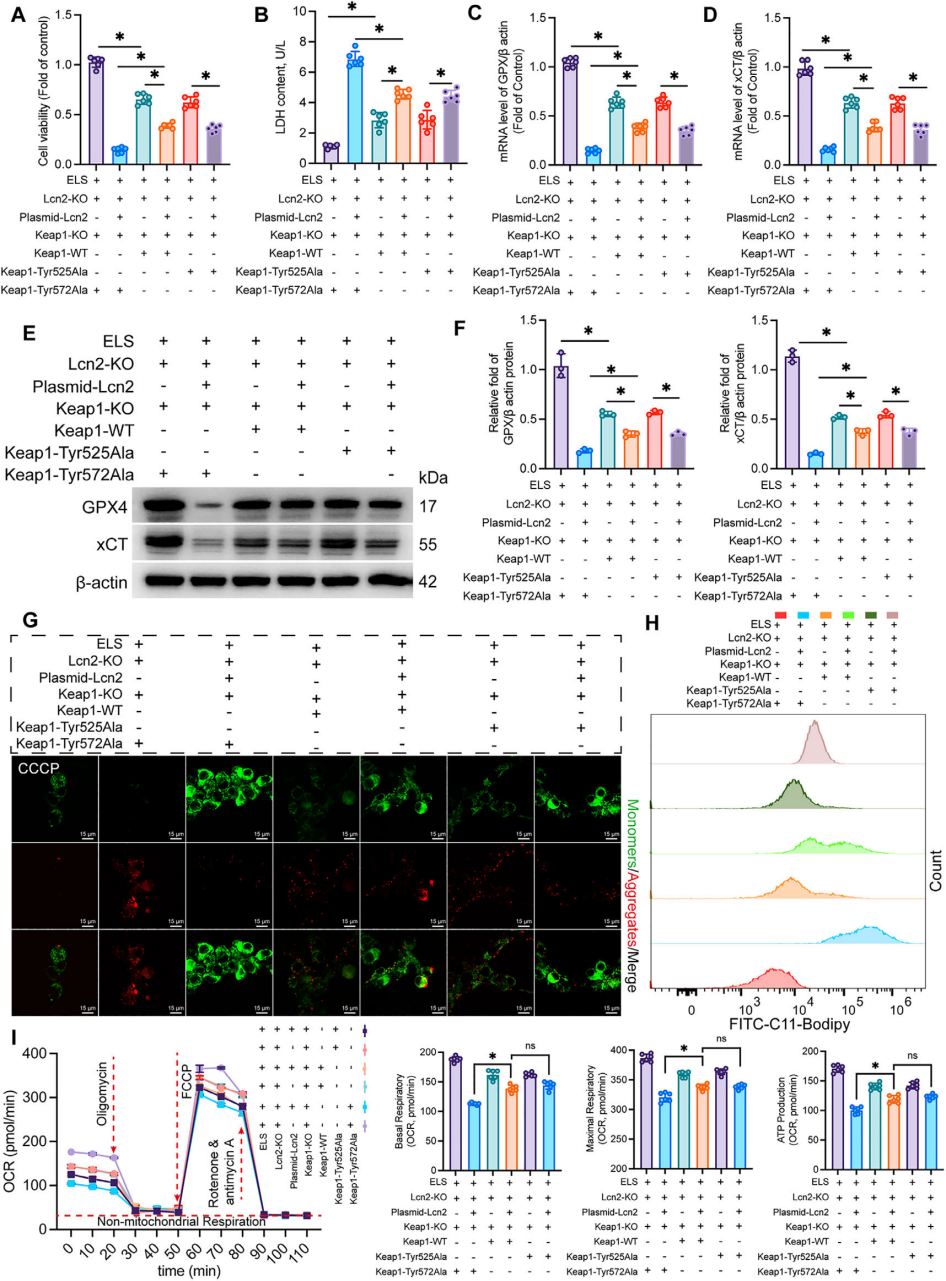
Keap1 mutations at the Tyr572 site inhibited the competitive binding of Lcn2 and promoted oxidative stress and mitochondrial function. (A) The CCK‐8 assay is used for detecting cell viability. *F* (5, 30) = 308.00, *p <* 0.0001. (B) LDH release level detection. *F* (5, 30) = 124.40, *p <* 0.0001. (C) Expression levels of GPX4 mRNA. *F* (5, 30) = 283.50, *p <* 0.0001. (D) Expression levels of xCT mRNA. *F* (5, 30) = 175.40, *p <* 0.0001. (E) Expression levels of GPX4 and xCT protein. (F) Quantification of results in Panel E. GPX4: *F* (5, 12) = 92.69, *p <* 0.0001. xCT: *F* (5, 12) = 266.70, *p* < 0.0001. (G) JC‐1 is used to detect mitochondrial membrane potential levels, with CCCP‐treated cells serving as a positive control. (H) C‐11 Bodipy flow cytometry is used to assess lipid peroxidation levels. I The Seahorse experiment is used to detect mitochondrial function. Basal respiratory: *F* (5, 30) = 135.20, *p* < 0.0001. Maximal respiratory: *F* (5, 30) = 107.40, *p* < 0.0001. ATP production: *F* (5, 30) = 110.80, *p* < 0.0001. The data were analyzed using one‐way analysis of variance and all data are expressed as the mean ± standard deviation. *p < 0.05 represents a statistically significant difference between the two groups. ns: no statistical difference.

We next tested the phenotypic consequences of Keap1 mutations. In Lcn2‑deficient neurons, Keap1 Tyr572Ala enhanced viability (Figure 8A), reduced LDH release (Figure 8B), upregulated xCT/GPX4 transcripts and proteins (Figure 8C–F), increased mitochondrial membrane potential (Figure 8G), lowered lipid peroxidation (C11‑BODIPY, MDA, 4‑HNE; Figure 8H; Figure S13A,B), boosted antioxidant capacity (GSH, SOD; Figure S13C,D), and decreased ROS and cellular iron (Figure S13E–G). Mitochondrial respiration and ATP production were concurrently elevated (Figure 8I). In contrast, in Lcn2‑replete neurons, Keap1 Tyr572Ala produced the opposite effects—dampened bioenergetics and heightened oxidative/ferroptotic stress. Tyr525 mutation behaved similarly to wild‑type Keap1 (Figure 8; Figure S13). These observations support a model in which Tyr572 is the critical competitive site for Lcn2, and its mutation benefits neurons only when Lcn2 is absent.

### 2.10 Keap1 Tyr572Ala Improves ICH Outcomes in Lcn2‑Deficient Mice but Worsens Disease When Lcn2 Is Present

We validated these context‑dependent effects in vivo. In Lcn2^fl/fl^Nestin^Cre^ mice, intraparenchymal delivery of Keap1 Tyr572Ala improved pathology and behavior compared with wild‑type Keap1: smaller hematoma, lower Longa and mNSS scores, longer rotarod latency, and reduced edema (Figure S14A–E). Conversely, in Lcn2^fl/−^Nestin^Cre^ mice, the same mutation aggravated ICH outcomes. Molecular analyses mirrored these trends: GPX4 and xCT transcripts and proteins tracked with protection (Figure S14F–H), apoptosis decreased in protected groups (Figure S14I), and iron deposition was reduced (Figure S14J–K). Cytokine profiling of peri‑hematomal tissue and serum showed that Tyr572Ala suppressed IL‑1β, TNF‑α, and IL‑18 while elevating IL‑10 and IL‑4 in Lcn2‑deficient mice, with the opposite pattern in Lcn2‑expressing mice (Figures S14L–O and Figure S15A,B). Similar to result in vitro, the effects observed with Keap1 WT and the Tyr525Ala mutant are comparable, whereas the Tyr572Ala mutation shows a distinct phenotype. This contrast further supports the conclusion that Tyr572 is a critical and specific regulatory site. Collectively, these data demonstrate that Keap1 Tyr572Ala exerts bidirectional, Lcn2‑dependent effects on ICH, conferring benefit only when Lcn2 is absent.

## 3 Discussion

This study identifies Lcn2 as a context‑dependent regulator of ferroptotic injury after ICH and reveals a mechanistic crosstalk with the Keap1‐Nrf2 antioxidant axis that governs neuronal fate. We show that LCN2 is markedly enriched within human hematoma relative to paired arterial blood, link neuronal Lcn2 deletion to improved functional and histological outcomes in ICH, and demonstrate that ferroptosis is attenuated via restoration of mitochondrial bioenergetics and suppression of lipid peroxidation. Mechanistically, Lcn2 competes with Nrf2 for binding to Keap1 at Tyr572, thereby restricting Nrf2 nuclear translocation and antioxidant transcriptional programs. A single‑site Keap1 mutation (Tyr572Ala) acts as a molecular switch whose effects invert with Lcn2 status: protective in Lcn2 deficiency, deleterious when Lcn2 is present. These findings refine current models of redox control in hemorrhagic brain injury and suggest biomarker‑guided strategies for precise modulation of ferroptosis.

LCN2 is a hematoma‑enriched driver of secondary injury [23, 24]. The robust intralesional accumulation of LCN2 in patients with ICH, validated across two independent cohorts, positions LCN2 as a candidate effector of peri‑hematomal neurotoxicity. Multi‑omics analyses in mice converge on ferroptosis and inflammatory signaling as the principal pathways altered by neuronal Lcn2. Together with the observation that exogenous Lcn2 reverses the benefits of neuronal knockout, these data support a model in which Lcn2 produced in or recruited to the hematoma compartment licenses lipid peroxidation, iron dysregulation, and inflammatory amplification in neighboring tissue. In addition, Lcn2 in human pathological samples is likely derived from multiple cellular sources. In addition to neurons, Lcn2 can be robustly produced by non‐neuronal cells, including activated microglia [25], astrocytes [26], and infiltrating immune cells (e.g., neutrophils and macrophages) [25, 27], particularly in the context of hemorrhagic injury. Therefore, the high levels of Lcn2 detected in the hematoma region may predominantly reflect contributions from these non‐neuronal sources. Importantly, we believe that these observations are not contradictory but rather complementary. Our mouse data reveal a cell‐autonomous, pathogenic role of neuron‐derived Lcn2, especially at early stages or in regions where neurons are still present. In contrast, in human lesions, particularly in areas with extensive neuronal loss, the sustained elevation of Lcn2 may be driven by glial and immune cells, potentially contributing to a broader inflammatory microenvironment. Thus, Lcn2 may function as a context‐dependent mediator, with both neuron‐derived and non‐neuronal Lcn2 participating in disease progression at different stages or anatomical regions.

A Keap1‐Nrf2 “gate” controlled by Lcn2 [28]. Nrf2 classically protects against ferroptosis by inducing GPX4, SLC7A11/xCT, and broader antioxidant and iron‑handling programs [29, 30]. Our work uncovers a previously unappreciated competitive interaction at Keap1 Tyr572. Lcn2 occupancy favors Nrf2 sequestration in the cytosol, whereas loss of Lcn2 or Tyr572Ala mutation disengages the brake and permits Nrf2 nuclear entry. This competition explains several key observations: Lcn2 knockout increases nuclear Nrf2 and upregulates anti‑ferroptotic effectors, reducing iron load and lipid peroxidation while restoring mitochondrial function. However, molecular docking predicted the binding interface between Lcn2 and Keap1, whose reliability is supported by the subsequent Co‐IP experiments. It could be added that future studies involving point mutations (e.g., in potential binding sites of Lcn2) or structural biology methods could provide more direct evidence. In addition, although Lcn2 deficiency leads to an increase in total Keap1 and Nrf2 protein levels, our Co‐IP results show a reduction in Keap1‐Nrf2 binding. This apparent discrepancy suggests that Lcn2 may play an active role in facilitating or stabilizing the Keap1‐Nrf2 complex, rather than merely competing for binding. We speculate that, on the one hand, the binding of Lcn2 to Keap1 may promote a conformation that is more favorable for Nrf2 recruitment. In the absence of Lcn2, Keap1 may adopt a conformation with reduced affinity for Nrf2, leading to decreased complex formation despite an increase in Keap1 abundance. Lcn2 may also influence the homeostasis of Keap1 protein. The observed increase in total Keap1 upon Lcn2 loss may reflect impaired turnover; however, the accumulated Keap1 could be functionally compromised or mislocalized, thereby reducing its effective interaction with Nrf2. On the other hand, Lcn2 may facilitate the assembly or activity of the Keap1‐Cul3 E3 ligase complex. Loss of Lcn2 could impair Nrf2 ubiquitination, resulting in Nrf2 accumulation, while simultaneously reducing detectable Keap1‐Nrf2 binding due to decreased complex cycling. Additionally, it is also possible that Lcn2 regulates the selective autophagy of Keap1 (e.g., via p62/SQSTM1). Altered autophagic flux may contribute to increased Keap1 levels while changing the dynamics of its interaction with Nrf2.

Genetic or pharmacologic Nrf2 inhibition (HY‑149508) abrogates the neuronal and metabolic rescue conferred by Lcn2 loss, confirming pathway dependency. The Tyr572Ala allele produces opposite phenotypes depending on Lcn2 abundance, resolving why universal “Nrf2 activation” may yield inconsistent efficacy across pathological contexts. These insights elevate Keap1 Tyr572 from a structural curiosity to a functional node that integrates extracellular inflammatory/iron signals (via Lcn2) with intracellular redox control (via Nrf2). In addition, Dual luciferase assay showed that Nrf2 directly transactivates GPX4, xCT, GSH, SOD, and IL‐10 promoters, but has no significant direct effect on transcriptional activation of pro‐inflammatory genes IL‑1β, TNF‑α, NOX, LOX, and IL‑18. This suggests that Nrf2's inhibition of these factors may occur indirectly. Accumulating evidence suggests that Nrf2 may exert anti‐inflammatory effects not only through direct transcriptional activation of antioxidant genes but also by modulating upstream inflammatory signaling pathways. One plausible mechanism is the suppression of NF‐κB signaling, which has been extensively reported in previous studies. Activation of Nrf2 can interfere with NF‐κB activity through multiple mechanisms [31, 32], including competition for transcriptional coactivators [33] (such as CBP/p300), upregulation of antioxidant enzymes that reduce ROS‐mediated NF‐κB activation, and stabilization of inhibitory proteins like IκB [34]. In this context, it is reasonable to speculate that Nrf2 activation may indirectly attenuate the expression of pro‐inflammatory cytokines by dampening NF‐κB signaling. Given that many of the inflammatory mediators observed in our study are canonical NF‐κB target genes, this regulatory axis may provide a mechanistic link between Nrf2 activation and the observed anti‐inflammatory effects.

The striking enrichment of LCN2 in hematoma suggests that LCN2 levels could guide selection or timing of therapies aimed at Keap1‐Nrf2. In LCN2‑high lesions, agents that merely boost Nrf2 may be insufficient unless Keap1 occupancy by LCN2 is relieved. Small molecules or peptides that disrupt the Lcn2‐Keap1 interaction at Tyr572, or that functionally emulate the beneficial effect of Tyr572Ala specifically in Lcn2‑deficient settings, could provide precision control of Nrf2 without global pathway activation. Our research proves that it is beneficial for a cell to have a “Lcn2‐sensor” on Keap1. A plausible advantage is that Lcn2 could act as a context‐sensitive modulator linking extracellular iron load and inflammatory cues to intracellular redox control. By sensing Lcn2, Keap1 may fine‐tune Nrf2 activation, enabling cells to dynamically adjust antioxidant responses to fluctuations in iron availability and inflammation intensity, thereby maintaining redox homeostasis more precisely. Given the intertwined roles of iron handling and inflammation, therapies that pair targeted modulation of the Lcn2–Keap1–Nrf2 interface with iron chelation or anti‑inflammatory agents may achieve additive or synergistic protection.

Prior studies have implicated Nrf2 activation as protective in ICH models [35, 36], yet the upstream determinants of Nrf2 availability have remained incompletely defined. Our data add a mechanistic layer by showing that Lcn2 can serve as an endogenous competitor for Keap1 binding, biasing the system toward ferroptosis. This framework also helps reconcile the literature reporting dual roles for Lcn2 in CNS injury [37]. Where Lcn2 is abundant and gains effective access to Keap1, it is poised to suppress Nrf2 and potentiate ferroptosis; in contexts with limited Lcn2 or distinct cellular sources, Keap1 may be freer to release Nrf2, enabling protection.

Several questions remain. First, the structural basis of Lcn2‐Keap1 engagement at Tyr572 warrants high‑resolution validation (e.g., cryo‑EM or co‑crystal structures) to guide rational drug design. Second, the cellular origins and spatial gradients of Lcn2 within hematoma and peri‑hematomal regions need to be mapped at single‑cell and subcellular resolution across time to define therapeutic windows. Third, while neuronal Lcn2 was central in this study, contributions from microglia [26, 38], astrocytes [26, 39], and endothelial cells [40, 41] likely shape the net redox environment and should be integrated into systems‑level models. Finally, long‑term outcomes and reparative processes (e.g., remyelination and synaptic plasticity) under selective modulation of the Lcn2‐Keap1‐Nrf2 axis require evaluation to ensure durable benefit and the larger, well‐characterized cohorts are necessary to validate the potential of LCN2 as a biomarker.

## 4 Conclusion

We propose that Lcn2 functions as a ligand‑level regulator of the Keap1‐Nrf2 gate at Tyr572, shifting the balance between ferroptosis and antioxidant defense after ICH. Neuronal deletion of Lcn2 disinhibits Nrf2, limits iron‑driven lipid peroxidation, preserves mitochondrial function, and improves neurological outcomes. The context‑dependent behavior of Keap1 Tyr572Ala underscores the need for biomarker‑guided targeting of this interface. These insights establish a mechanistic rationale for precision therapeutics that modulate the Lcn2/Keap1/Nrf2 axis to mitigate ferroptotic injury in ICH.

## 5 Materials and Methods

### 5.1 Lentivirus and Plasmids

Cells/mice were infected with lentivirus/adeno‐associated viruses (AAVs) to stably knockdown Nrf‐2 (Nrf‐2‐KD). The lentivirus/AAVs was constructed with the assistance of GeneChem Co., Inc. (Shanghai, China). Lcn2 and Keap1 were knocked out using CRISPR/Cas9 technology. Cas9 and single‐guide RNA (sgRNA) lentiviruses were designed and constructed by GeneChem Co., Ltd. (Shanghai, China). Cell lines were screened with puromycin. All cell lines used in this study have undergone authentication by short tandem repeat (STR) analysis to confirm their identity and tested mycoplasma free.

Nrf‐2 overexpression plasmid, Lcn2 overexpression plasmid, Keap1 overexpression plasmid, Keap1 mutant plasmid (Tyr525Ala and Tyr572Ala), luciferase reporter plasmid (RV‐GPX4, xCT, GSH, SOD, IL‐10, IL‐1β, TNF‐α, IL‐18, Nox, and Lox), pcDNA3.1, and negative control (NC, luciferase reporter plasmid without promoter) were designed and constructed by Hanbio Co., Ltd. (Shanghai, China). Plasmid transfection was performed using jetPRIME DNA transfection reagent (PolyPlus) according to the manufacturer's instructions.

### 5.2 Mouse Cytokine Array

The brain tissue around the bleeding site was used for the detection of inflammatory factors and cytokines. The mouse cytokine 2808 array was purchased from Ray‐Biotech (AAM‐BLG‐2808; USA). The experimental procedures were performed in strict accordance with the manufacturer's instructions.

### 5.3 Proteomics Testing and Analysis

The proteomics detection steps, including sample preparation, LC‐MS/MS analysis, and database search, are described in the Supporting Information Materials.

### 5.4 Dual‐Luciferase Reporter Assay

Firefly luciferase reporter vector (RV: including GPX4, xCT, GSH, SOD, IL‐10, IL‐1β, TNF‐α, IL‐18, Nox, and Lox) and Renilla reporter plasmids and Nrf‐2 overexpression plasmid (pcDNA3.1 as control) were transfected into each modified cell line. The reporter vector without corresponding gene promoter was used as NC. The cells were cultured for 24 h after plasmid transfection, and the fluorescence intensity of each treatment group was detected using the Dual‐Luciferase Reporter Assay Kit (Promega).

ICH modeling, Longa score, modified Neurological Severity Score (mNSS), Rotarod test, co‐immunoprecipitation (Co‐IP), western blot (WB), quantitative PCR (qPCR), enzyme‐linked immunosorbent assay (Elisa), immunofluorescence (IF), transmission electron microscopy (TEM), hematoxylin and eosin (H&E) staining, and flow cytometric detection of apoptosis

All the detailed experimental procedures described above were carried out in accordance with the descriptions in our team's previously published research findings [10, 42, 43, 44, 45, 46, 47]. Regarding the dosing regimen in animal experiments, the details are as follows: when utilizing a mouse model of ICH with autologous blood, Lcn2 protein is administered simultaneously at a dosage of 1 µg per 25 g of body weight.

Detailed information on the qPCR primers can be found in Table S2. The company and catalog number information for antibodies, ELISA kits (including IL‐1β, TNF‐α, IL‐18, IL‐4, IL‐10, and 4‐HNE), flow cytometric apoptosis detection kit and other reagent is provided in Table S3.

About ICH model, in our prior study [10, 20, 21, 22], Lcn2 expression is markedly upregulated and reaches a peak (or near‐peak) level around Day 3 post‐ICH. Neuroinflammation and perihematomal edema are also prominent at this stage. This time point represents a critical phase of secondary brain injury, during which inflammatory responses, oxidative stress, and cell death processes are highly active. Based on these observations, post‐ICH Day 3 was selected as a representative and biologically relevant time point to capture robust molecular changes and functional deficits associated with Lcn2.

Mouse genotype detection, measurement of brain edema content, mitochondrial membrane potential detection, separation of cytoplasmic and nuclear proteins, GSH, SOD, and MDA detection, C11 BODIPY lipid peroxidation detection, ferrous ion content detection, Agilent Seahorse XF cell mito stress test assay, and extraction and cultivation of primary neurons

All the detailed experimental procedures described above are described in the Supporting Information Materials.

### 5.5 Data Processing

As described in our previously published research [48], molecular biology experiments were conducted using three technical replicates for each mouse, and the results were averaged. For each group, three mice were included, and the mean value from each mouse was used for statistical analysis (*n* = 3 per group). For in vitro experiments, cells were seeded in three wells for each assay, and the average value from these wells was calculated. Each experiment was independently repeated three times as biological replicates. For pathological analyses, one tissue section from each of three mice per group was examined using immunofluorescence laser confocal microscopy. Three random fields were selected from each section for quantitative analysis of the measured indicators, and the values were averaged. The mean values obtained from the three mice (*n* = 3 per group) were then used for statistical comparisons between groups.

### 5.6 Statistical Analysis

Statistical analyses were performed using Prism 8 for macOS. Power analysis was conducted using PASS software to determine appropriate sample sizes. A power value greater than 0.9 was considered sufficient to ensure adequate statistical power in the experimental design. All data are presented as mean ± standard deviation (SD). Depending on the homogeneity of variance, either parametric or nonparametric tests were applied. For comparisons between groups, Student's *t*‐test or one‐way analysis of variance (ANOVA) was used as appropriate, followed by Sidak's or Tukey's multiple comparisons test. *p* value < 0.05 was considered statistically significant.

## Author Contributions

D.Y.N., H.H.K., and L.Q. designed the study, performed the experiments, and prepared the manuscript, and they contributed equally to this work. W.G.M., H.L.J., L.J.B., Z.B.Y., L.Y., H.G.H., W.L., Z.F., and X.L. were involved in experiment performance and data collection. F.X.W., J.L., and W.J.L. were responsible for the supervision of the entire project and were involved in the study design, data interpretation, manuscript preparation, and funding. All authors read and approved the final manuscript.

## Funding

This study was supported by the China Postdoctoral Science Foundation, No.18 Special Fund (2025T181193, FXW) and the National Natural Science Foundation of China (82571483 to W.J.L. and 82401720 to D.Y.N.).

## Conflicts of Interest

The authors declare no conflicts of interest.

## Ethics Statement

Human ethical approval was obtained from the Xijing Hospital Research Ethics Committee (KY20252031‐C‐1), and written informed consent was obtained from each patient. Patients with ICH who underwent surgery within 24 h of symptom onset were included. After induction of general anesthesia and prior to surgery, 1 mL of arterial blood was collected via radial artery puncture from the same patient, and hematoma samples removed during surgery were also collected for measurement of LCN2 levels in arterial blood. All samples were immediately snap‐frozen in liquid nitrogen after collection. All animal experiments were performed in accordance with protocols approved by the Institutional Ethics Committee of Xijing Hospital. All animal experimental procedures were approved by the Institutional Animal Care and Use Committee of Air Force Military Medical University (IACUC: 20210424). The ARRIVE 2.0 guidelines were followed for animal data report. Male Lcn2^fl/−^Nestin^Cre^ and Lcn2^fl/fl^Nestin^Cre^ mice were purchased from Cyagen Biotechnology Co., Ltd (Jiangsu, China). All mice were maintained in the same environment. Moreover, this study was performed in accordance with the principles of the Declaration of Helsinki.

## Supporting information




**Supporting File 1**: mco270920‐sup‐0001‐SuppMat.docx

**Supporting File 2**: mco270920‐sup‐0002‐Figure3A.xls

**Supporting File 3**: mco270920‐sup‐0003‐Figure3E.xlsx

## Data Availability

The data that support the findings of this study are available from the corresponding author upon reasonable request.
