# Supplementary material for: Lcn2 Promotes Ferroptosis in Intracerebral Hemorrhage by Targeting Keap1 Tyr572 and Restricting Nrf2 Activation
Source: MedComm (2020). 2026 Aug 16;7(9):e70920. doi: 10.1002/mco2.70920 (PMC13478554; doi:10.1002/mco2.70920)
Supplement: Supplementary file 1 [file MCO2-7-e70920-s003.docx]

**Bidirectional control of ferroptosis by Keap1 Tyr572Ala reveals Lcn2‑dependent redox gating in intracerebral hemorrhage**

Ya-nan Dou^1#^, Hongkang Hu^2#^, Qinghua Li^3#^, Guangming Wang^2^, Lijun Hou^2^, Junbin Liu^4^, Buyi Zheng^5^, Ying Li^6^, Guohan Hu^2^, Zhou Fei^1^, Xia Li^1^, Li Wang^1^, Jialiang Wei^1*^, Lei Jiang^2*^, Xiaowei Fei^1*^

^1^Department of Neurosurgery, Xijing Hospital, Air Force Military Medical University, Xi'an, Shaanxi, 710032, China.

^2^Department of Neurosurgery, Changzheng Hospital, Naval Medical University, Shanghai, 200003, China.

^3^Department of Pediatrics, Shanghai Ninth People’s Hospital, Shanghai JiaoTong University School of Medicine, Shanghai, 200023, China.

^4^Department of Orthopedics, Changzheng Hospital, Naval Medical University, Shanghai, 200003, China

^5^Department of Neurosurgery, Wenzhou People’s Hospital, Wenzhou, Zhejiang, 325000, China.

^6^Department of Pathology, Changzheng Hospital, Naval Medical University, Shanghai, 200003, China.

#These authors contributed equally to this work.

^*^Correspondence should be addressed to Jialiang Wei (weijl@fmmu.edu.cn), Lei Jiang (jiangleicz@smmu.edu.cn) and Xiaowei Fei (xiaowei_fei@fmmu.edu.cn).

**Materials and methods**

**Mouse genotype detection**

Mouse tails were cut, digested with proteinase K for 20 min at 55 °C, and further inactivated with proteinase K for 5 min at 100 °C. Polymerase chain reaction (PCR) was performed according to the protocol of the One Step Mouse Genotyping Kit (Vazyme, China). Lcn2 primers: F: 5’-TGA TCA TTC TGT GTC CTA GGG GAT G-3’, R: 5’-TTA GCC TCT TCC AAG GCT AGA CAA-3’ (Homozygotes: one band with 203 bp, Heterozygotes: two bands with 203 bp and 144 bp, Wildtype allele: one band with 144 bp). Nestin-Cre primers: P1: 5’-TTG CTA AAG CGC TAC ATA GGA-3’, P2: 5’-GCC TTA TTG TGG AAG GAC TG-3’, P3: 5’-CCT TCC TGA AGC AGT AGA GCA-3’ (Mutant: 150bp, Wild type: 246bp).

**Measurement of brain edema content**

After the mice are euthanized, the brain tissue is quickly removed and gently blotted dry with filter paper. Its wet weight (W_wet) is then measured on a tared weighing dish. The tissue is subsequently placed in a constant-temperature drying oven at 105°C and dried for 24 hours or until its weight remains constant. It is then cooled to room temperature and weighed again to obtain the dry weight (W_dry). Finally, the brain water content is calculated using [(W_wet - W_dry) / W_wet] × 100%, and the differences among various treatments or groups are compared.

**Mitochondrial membrane potential detection**

Use the Mitochondrial Membrane Potential Assay Kit (JC-1) (Beyotime, C2006) to measure mitochondrial membrane potential. For adherent cells in a six-well plate, remove the culture medium, wash the cells once with PBS, and add 1 ml of cell culture medium. The cell culture medium may contain serum and phenol red. Add 1 ml of JC-1 staining working solution and mix thoroughly. Incubate at 37 °C in a cell incubator for 20 min. During incubation, prepare an appropriate volume of JC-1 staining buffer (1X) by mixing 1 ml of JC-1 staining buffer (5X) with 4 ml of distilled water, and place it in an ice bath. After the 20-min incubation at 37 °C, remove the supernatant and wash the cells twice with JC-1 staining buffer (1X). Add 2 ml of cell culture medium, which may contain serum and phenol red. Observe the cells under a fluorescence microscope or laser confocal microscope. For detecting JC-1 monomers, set the excitation wavelength to 490 nm and the emission wavelength to 530 nm; for detecting JC-1 aggregates, set the excitation wavelength to 525 nm and the emission wavelength to 590 nm.

**Separation of cytoplasmic and nuclear proteins**

To separate nuclear and cytoplasmic proteins, a nuclear and cytoplasmic protein extraction kit (Beyotime, P0027) was used, following the kit instructions precisely. In brief, for adherent cells, wash once with PBS, then use a cell scraper to detach the cells or treat them with EDTA solution to loosen their attachment, and gently dislodge them with a pipette. Collect the cells by centrifugation, remove the supernatant as thoroughly as possible, and retain the cell pellet. For every 20 μL of cell pellet, add 200 μL of PMSF-supplemented cytoplasmic protein extraction reagent A. Vortex at maximum speed for 5 seconds to fully suspend and disperse the pellet, then place the sample on ice for 10-15 min. Next, add 10 μL of cytoplasmic protein extraction reagent B, vortex at maximum speed for 5 seconds, place on ice for 1 min and vortex again at maximum speed for 5 seconds. Centrifuge at 12,000-16,000 × g for 5 min at 4 °C. Immediately transfer the supernatant to a prechilled plastic tube; this supernatant is the extracted cytoplasmic protein. For the pellet, remove any residual supernatant and add 50 μL of PMSF-supplemented nuclear protein extraction reagent. Vortex at maximum speed for 15-30 seconds to fully suspend and disperse the pellet. Return the sample to ice, and every 1-2 minutes vortex again at high speed for 15-30 seconds, over a total of 30 minutes. Finally, centrifuge at 12,000-16,000 × g for 10 min at 4 °C. Immediately transfer the supernatant to a prechilled plastic tube; this supernatant is the extracted nuclear protein.

**GSH, SOD and MDA Detection**

Use the reduced glutathione (GSH) content assay kit (Solarbio, BC1175), SOD activity assay kit (Solarbio, BC5165) and Malondialdehyde (MDA) content assay kit (Solarbio, BC0025) to measure the GSH content, SOD activity and MDA content in each group of samples. Add the standards and samples according to the table and dosages specified in the kit instructions. All experimental steps were strictly performed according to the kit instructions.

**C11 BODIPY lipid peroxidation detection**

Dissolve 1 mg of the lipid-soluble, ratio-type fluorescent probe C11 BODIPY 581/591 (ABclonal, RM02821) in 198.2 μL of DMSO to prepare a 10 mM stock solution of C11 BODIPY 581/591. Culture the target cells to an appropriate density, perform the necessary treatments to establish the relevant cell model, then add the appropriate volume of C11 BODIPY 581/591 to achieve a final concentration of 5 μM and incubate for 1 hour. Wash the cells twice with PBS to remove excess dye. Use a flow cytometer with single-channel detection, excite with a 488 nm laser, and measure the signal in the 505-550 nm range.

**Ferrous ion content detection**

Using the ferrous ion content detection kit (Solarbio, BC5415) to determine the iron ion content in each sample group. Homogenize the samples on ice according to the ratio of tissue mass (g) to extraction solution volume (mL) of 1:5-10. Centrifuge at 10,000 g and 4 °C for 10 min, then collect the supernatant and place it on ice for measurement. Preheat the visible spectrophotometer/microplate reader for more than 30 min, set the wavelength to 593 nm, and zero the spectrophotometer with distilled water. The volume and order of sample addition to each well should strictly follow the table in the kit manual. Vortex thoroughly for 5 min, then centrifuge at 12,000 g at room temperature for 10 min. Carefully transfer 200 μL of the upper inorganic phase to a micro glass cuvette or 96-well plate, measure the absorbance at 593 nm (denoted as A_measured), and calculate ΔA_measured = A_measured - A_blank. Similarly, take 200 μL to measure the absorbance at 593 nm (denoted as A_standard and A_blank) and calculate ΔA_standard = A_standard - A_blank. Based on the standard tube concentration (x, μmol/L) and the absorbance ΔA_standard (y, ΔA_standard), establish a standard curve. Using this standard curve, substitute ΔA_measured (y, ΔA_measured) to determine the sample concentration (x, μmol/L). Then, express it in terms of the protein concentration: ferrous ion content (μmol/mg prot) = x × 10³ × V_extraction ÷ (C_pr × V_extraction) = 0.001x ÷ C_pr. Here, C_pr is the protein concentration (mg/mL), and V_extraction is the volume of extraction solution added (1 mL).

**Agilent Seahorse XF cell mito stress test assay**

Experimental cells are seeded into Seahorse-specific microplates at an appropriate density according to the experimental design, allowing them to adhere and grow. The requisite measurement reagents (such as oligomycin, FCCP, and rotenone/antimycin) are added separately to the injection ports of the Seahorse sensor cartridge, and the sensors are rinsed and calibrated in a prepared calibration solution. Next, the culture plate containing the adhered cells and the calibrated sensor cartridge are placed into the Agilent Seahorse XFe96 analyzer (Agilent Technologies, Santa Clara, CA, USA), where the reagents are sequentially injected into each well according to the preset program, enabling real-time detection of oxygen consumption rate (OCR) basal respiratory, maximal respiratory and ATP production respiratory. Finally, the collected data are analyzed and processed using Seahorse XF Cell Mito Stress Test Report Generator package to assess mitochondrial function, glycolytic capacity, or the overall energy metabolic status.

**Extraction and cultivation of primary neurons**

Neonatal C57BL/6 J mice were used to extract primary neurons using a stereomicroscope. The culture dish was coated with 0.2 mg/mL of poly-L-lysine (Sigma-Aldrich) overnight at 37 °C, washed three times with sterile water and placed in an incubator for use. The brain tissue was minced with sterile ophthalmic scissors, digested with 0.25% trypsin for 5 min at 37 °C before the brain tissue was centrifuged at 1000 rpm for 5 min. For the extraction of primary neurons, the complete Dulbecco’s modified Eagle’s medium (DMEM, Gibco) was used for appropriate dilution and the cell suspension was made into a seed plate. After 4-6 h, DMEM was replaced with Neurobasal medium (Gibco) for primary neurons which containing 0.25% glutamine (Sigma-Aldrich), 1% penicillin/streptomycin (Gibco) and 2% B-27 supplement (Gibco). Neurons monolayers were obtained at the bottom of the dish after 7 days. The neurons were identified by morphological analysis and NeuN/MAP2/β-tubilin-III staining.

**Proteomics testing and analysis**

**Sample Preparation**

The samples were placed on ice for re -melting, samples were rinsed using PBS, and 200 ul of strong RIPA (Thermo Scientific, 89900) was added to 20 mg of brain tissue. Grinding was performed with a tissue grinder after adding grinding beads, frequency: 65Hz, run for 30s, stop for 10s, and repeat 2 times. The lysis mixture was lysed on ice for 30 min. 4°C, centrifuged at 10,000g for 10 min and the supernatant was collected. Add 5 times the volume of acetone to the supernatant, precipitate for 1h at -80°C, centrifuge for 10min at 4°C, 10,000g. Wash the precipitate twice with 80% acetone, centrifuge f or 10min at 4°C, 10,000g, and discard the supernatant. Next, the protein precipitate was sonicated with 50 mM NH4 HCO3 to re-solubilize the protein. Protein concentration was determined using a BCA kit (beyotime, P0012), and trypsin (promega, V5113) was added at a 50:1 ratio and digested at 37°C for 12-16 h. A second digestion was then performed by adding a 100:1 addition of trypsin solution for 2 h. The digested peptides were digested using a C18 Cartridge (Waters The peptides were desalted using a C18 Cartridge (Waters, WAT054955) and subsequently eluted with 70% acetonitrile and 0.1% formic acid solution, and the eluate was vacuum lyophilized. 0.1% formic acid solution was used to re -solubilize the lyophilized peptides, and the peptide concentration was de termined using a Pierce Quantitative Colorimetric Peptide Assay (Thermo Scientific, 23275) kit. The peptide concentration was determined using Pierce Quantitative Colorimetric Peptide Assay (Thermo Scientific, 23275). The peptides were graded into 30 fract ions using reverse liquid chromatography, lyophilized under vacuum, and then rehydrated with 0.1% formic acid in preparation for the machine.

**LC-MS/MS Analysis**

The sample peptides were chromatographically separated by an EASY-nLC 1200nl-class liquid chromatography system (Thermo Scientific) and detected using an on -line Q Exactive HF-X mass spectrometer (Thermo Scientific). The mobile phase A was 0.1% formic acid aqueous solution, and the mobile phase B was 0.1% formic acid acetonitrile aqueous solution (80% acetonitrile). The chromatographic column was equilibrated with 100% of liquid A. The sample was separated by a reversed-phase C18 column (75 μm × 15 cm, Reprosil-Pur C18, 1.9 μm) with a gradient at a flow rate of 350 nl/min. The elution gradient was as follows: peptides were separated and analyzed by DDA data-dependent acquisition mode mass spectrometry using a Q Exactive HF-X mass spectrometer with positive ion detection mode. Parent ion scan range: 350-2000 m/z, primary MS1 resolution: 60000 @ m/z 200, AGC target: 3 × 106, Maximum IT: 50 ms. secondary MS2 acquisition mode: HCD fragmentation of the top 20 parent ions in the primary spectrum after each full scan (full scan) The corresponding secondary mass spectra (MS2 scan) are acquired. Secondary mass spectrometry resolution: 15000 @ m/z 200, AGC target: 1 × 10 5, Maximum IT: 45 ms, HCD collision energy: 28%, dynamic exclusion time: 30s.

**Database Search**

The mass spectrometry offline RAW files were imported into MaxQuant (v2.3.1.0) software for proteomic database search. The database used for the search was obtained from the uniprot website (https://www.uniprot.org/).

Supplemental Table 1. Baseline clinical characteristics of patients with intracerebral hemorrhage

| Variable | Overall (n = 30) | Correlating LCN2 ratio with … |
| --- | --- | --- |
| Arterial blood LCN2 | 85.40 (51.90, 104.33) |  |
| Hematoma LCN2 | 1037.45 (544.30, 1979.25) |  |
| Hematoma volume, mL | 14.2 (6.9, 27.5) | Spearman rho (0.996), P = 0.00 |
| GCS score | 11 (7, 14) | Spearman rho (-0.998), P = 0.00 |
| LCN2 ratio | 59.07 (19.06, 95.35) |  |
| Hemorrhage location |  | Kruskal-Wallis P = 0.508 |
| Basal ganglia | 9 (30.0%) |  |
| Lobar | 12 (40.0%) |  |
| Thalamus | 7 (23.3%) |  |
| Cerebellum | 2 (6.7%) |  |

*Note.*

Data are presented as median (IQR) or n (%). GCS, Glasgow Coma Scale; LCN2, lipocalin-2.

Supplemental Table 2. The primer sequence for qPCR

| Primer | Sequence（5' to 3'） |
| --- | --- |
| Lcn2-mus-F | 5'-GCAGGTGGTACGTTGTGG-3' |
| Lcn2-mus-R | 5'-GTTGTAGTCCGTGGTGGC-3' |
| GPX4-mus-F | 5'-TGTGCATCCCGCGATGATT-3' |
| GPX4-mus-R | 5'-CCCTGTACTTATCCAGGCAGA-3' |
| xCT-mus-F | 5'-GGCACCGTCATCGGATCAG-3' |
| xCT-mus-R | 5'-CTCCACAGGCAGACCAGAAAA-3' |
| GAPDH-mus-F | 5'-AGGTCGGTGTGAACGGATTTG-3' |
| GAPDH-mus-R | 5'-GGGGTCGTTGATGGCAACA-3' |

Supplemental Table 3. Details of antibodies and reagents

| Reagent | Company | Art.No. |
| --- | --- | --- |
| Anti-NeuN | abcam | ab177487 |
| Anti-MAP2 | abcam | ab254264 |
| Anti-β-Tubulin-III | abcam | ab18207 |
| Anti-GPX4 | abcam | ab125066 |
| Anti-xCT | abcam | ab216876 |
| Anti-β-actin | abcam | ab6276 |
| Anti-Nrf2 | CST | 12721 |
| Anti-H3 | abcam | ab1791 |
| Anti-Lcn2 | abcam | ab216462 |
| Anti-Keap1 | abcam | ab119403 |
| Anti-IgG | abcam | ab 172730 |
| Anti-Ferroportin | Proteintech | 26601-1-AP |
| Anti-Hepcidin | abcam | ab190775 |
| Annexin V-FITC | Beyotime | C1062S |
| IL-1β ELISA Kit | HUABIO | EM0001 |
| TNF-α ELISA Kit | HUABIO | EM0010 |
| IL-18 ELISA Kit | HUABIO | EM0034 |
| IL-4 ELISA Kit | HUABIO | EM0003 |
| IL-10 ELISA Kit | HUABIO | EM0005 |
| 4-HNE Assay Kit | abcam | ab238538 |
| Nrf2-IN-3 | MCE | HY-149508 |
| Lcn2 protein | abcam | ab282399 |
| Ferrostatin-1 | MCE | HY-100579 |
| RSL3 | MCE | HY-100218A |
| RhoNox-1 | MCE | HY-D1533 |

**S Figure 1. GO analysis results of differentially expressed gene.**

**A** Cell component analysis. **B** Biological process analysis. **C** Molecular function analysis.

**S Figure 2. Lcn2^fl/fl^Nestin^Cre^ mice exhibit lower levels of ferroptosis after ICH compared to Lcn2^fl/-^Nestin^Cre^ mice.**

**A** Expression levels of GPX4 mRNA. F (2, 12) = 38.21, *P < 0.0001*. **B** Expression levels of xCT mRNA. F (2, 12) = 65.52, *P < 0.0001*. **C** Expression levels of GPX4 and xCT protein. GPX4: F (2, 12) = 20.47, *P = 0.0001*. xCT: F (2, 12) = 23.52, *P < 0.0001*. **D** The immunofluorescence assay detected the co-localization levels of NeuN, GPX4, and xCT in the tissue surrounding the hemorrhage. **E** Iron ion staining. **F** Detection of iron ion content in brain tissue surrounding hemorrhage. F (2, 12) = 13.15, *P = 0.0009*. **G** Serum iron ion content detection. F (2, 12) = 35.19, *P < 0.0001*. **H** Detection of MDA levels. F (2, 12) = 18.12, *P = 0.0002*. **I** Detection of 4-HNE levels. F (2, 12) = 8.774, *P = 0.0045*. **J** Detection of GSH levels. F (2, 12) = 51.72, *P < 0.0001*. **K** Detection of SOD activity. F (2, 12) = 21.93, *P < 0.0001*. The data were analyzed using one‐way analysis of variance and all data are expressed as the mean ± standard deviation. **P < 0.05* represents a statistically significant difference between the two groups. ns: no statistical difference.

**S Figure 3. Expression of ferroportin and hepcidin in various groups of mice.**

**A** WB is used to detect the expression of Ferroportin and hepcidin in the surrounding tissues of various mouse hemorrhages. **B** Quantification of results in Panel A. Ferroportin: F (3, 8) = 36.18, *P < 0.0001*. Hepcidin: F (3, 8) = 67.80, *P < 0.0001*. The data were analyzed using one‐way analysis of variance and all data are expressed as the mean ± standard deviation. **P < 0.05* represents a statistically significant difference between the two groups.

**S Figure 4. Verification of Lcn2 knockout efficiency.**

**A** Expression of Lcn2 mRNA levels. t = 11.17, df = 8, P < 0.0001. **B** Expression of Lcn2 protein levels. **C** Quantification of results in Panel B. t = 12.18, df = 8, P < 0.0001. The data were analyzed using one‐way analysis of variance and all data are expressed as the mean ± standard deviation. **P < 0.05* represents a statistically significant difference between the two groups.

**S Figure 5. Lcn2 protein concentration screening for cell treatment.**

**A** The CCK experiment is used to detect the effects of different concentrations of Lcn2 protein treatment on cell viability. F (15, 64) = 1.650, *P = 0.0852*. The data were analyzed using one‐way analysis of variance and all data are expressed as the mean ± standard deviation. **P < 0.05* represents a statistically significant difference between the two groups.

**S Figure 6. Expression levels of GPX4 and xCT mRNA.**

**A** Expression levels of GPX4 mRNA. F (3, 16) = 33.71, *P < 0.0001*. **B** Expression levels of xCT mRNA. F (3, 16) = 26.24, *P < 0.0001*. The data were analyzed using one‐way analysis of variance and all data are expressed as the mean ± standard deviation. **P < 0.05* represents a statistically significant difference between the two groups.

**S Figure 7. Expression of ferroportin and hepcidin in various groups of mice.**

**A** WB is used to detect the expression of Ferroportin and hepcidin in cells with different treatment. **B** Quantification of results in Panel A. Ferroportin: F (3, 8) = 42.28, *P < 0.0001*. Hepcidin: F (3, 8) = 43.53, *P < 0.0001*. The data were analyzed using one‐way analysis of variance and all data are expressed as the mean ± standard deviation. **P < 0.05* represents a statistically significant difference between the two groups.

**S Figure 8. Ferroptosis inhibitor Ferrostain-1 promotes the upregulation of neuronal activity in ELS conditions.**

**A** The CCK-8 assay is used for detecting cell viability (1 μM Ferrostain-1 for 24 h). F (2, 12) = 303.2, *P < 0.0001*. **B** C-11 Bodipy flow cytometry is used to assess lipid peroxidation levels. **C** Quantification of results in Panel B. F (2, 12) = 23.64, *P < 0.0001*. The data were analyzed using one‐way analysis of variance and all data are expressed as the mean ± standard deviation. **P < 0.05* represents a statistically significant difference between the two groups.

**S Figure 9. Ferroptosis agonist RSL3 inhibits the recovery of cell viability and the reduction of lipid peroxidation induced by Lcn2-KO.**

**A** The CCK-8 assay is used for detecting cell viability (1 μM RSL3 for 24 h). F (2, 12) = 133.60, *P < 0.0001*. **B** C-11 Bodipy flow cytometry is used to assess lipid peroxidation levels. **C** Quantification of results in Panel B. F (2, 12) = 59.58, *P < 0.0001*. The data were analyzed using one‐way analysis of variance and all data are expressed as the mean ± standard deviation. **P < 0.05* represents a statistically significant difference between the two groups.

**S Figure 10. RhoNox-1 probe staining in each group.**

**A** Representative fluorescence image of RhoNox-1 probe staining**. B** Quantification of results in Panel A. F (3, 16) = 18.47, *P < 0.0001*. The data were analyzed using one‐way analysis of variance and all data are expressed as the mean ± standard deviation. **P < 0.05* represents a statistically significant difference between the two groups.

**S Figure 11. The impact of Nrf-2 knockdown on the prognosis of ICH in Lcn2^fl/-^Nestin^Cre^ mice.**

**A** Comparison of hemorrhagic areas in each group of mice. t = 10.54, df = 10, *P < 0.0001*. **B** Longa scores in each group of mice. t = 4.33, df = 18, *P = 0.0004*. **C** mNSS scores in each group of mice. t = 8.779, df = 18, *P < 0.0001*. **D** The rotarod test measures the duration that mice can maintain their balance on a rotating rod. t = 10.25, df = 18, *P < 0.0001*. **E** brain water content in each group of mice. t = 6.272, df = 18, *P < 0.0001*. **F** Assess the level of apoptosis in the brain tissue surrounding the hemorrhage in each group of mice using flow cytometry. **G** Quantification of results in panel F. t = 5.399, df = 4, *P = 0.0057*. **H** The Elisa test detected the expression levels of inflammation-related factors in the brain tissue surrounding the hemorrhage. IL-1β: t = 4.643, df = 10, *P = 0.0009*. TNF-α: t = 7.683, df = 10, *P < 0.0001*. IL-18: t = 11.09, df = 10, *P < 0.0001*. IL-4: t = 3.632, df = 10, *P = 0.0046*. IL-10: t = 5.466, df = 10, *P = 0.0003*. **I** The Elisa test detected the expression levels of inflammation-related factors in mice serum. IL-1β: t = 3.385, df = 10, *P = 0.0069*. TNF-α: t = 6.104, df = 10, *P = 0.0001*. IL-18: t = 6.987, df = 10, *P < 0.0001*. IL-4: t = 5.554, df = 10, *P = 0.0002*. IL-10: t = 7.626, df = 10, *P < 0.0001*. The data were analyzed using Student T test and all data are expressed as the mean ± standard deviation. **P < 0.05* represents a statistically significant difference between the two groups.

**S Figure 12. HY-149508 and Nrf2 knockdown inhibit Lcn2-induced mitochondrial function recovery and lipid peroxidation.**

**A** The CCK-8 assay is used for detecting cell viability. F (6, 35) = 103.3, *P < 0.0001*. **B** LDH release level detection. F (6, 35) = 27.83, *P < 0.0001*. **C** Expression levels of GPX4 and xCT mRNA. GPX4: F (6, 35) = 97.92, *P < 0.0001*. xCT: F (6, 35) = 140.60, *P < 0.0001*. **D** Detection of mitochondrial ROS expression levels. F (6, 35) = 283.1, *P < 0.0001*. **E** Detection of total ROS expression levels. F (6, 35) = 51.11, *P < 0.0001*. **F** Detection of cellular iron ion content. F (6, 35) = 39.10, *P < 0.0001*. **G** Detection of 4-HNE levels. F (6, 35) = 10.01, *P < 0.0001*. **H** Detection of SOD activity. F (6, 35) = 87.03, *P < 0.0001*. The data were analyzed using one‐way analysis of variance and all data are expressed as the mean ± standard deviation. **P < 0.05* represents a statistically significant difference between the two groups.

**S Figure 13. Keap1 mutations at the Tyr572 sites inhibited the competitive binding of Lcn2 and improved oxidative stress and mitochondrial function.**

**A** Detection of MDA levels. F (5, 30) = 85.20, *P < 0.0001*. **B** Detection of 4-HNE levels. F (5, 30) = 89.66, *P < 0.0001*. **C** Detection of GSH levels. F (5, 30) = 123.20, *P < 0.0001*. **D** Detection of SOD activity. F (5, 30) = 182.60, *P < 0.0001*. **E** Detection of mitochondrial ROS expression levels. F (5, 30) = 66.47, *P < 0.0001*. **F** Detection of total ROS expression levels. F (5, 30) = 52.22, *P < 0.0001*. **G** Detection of cellular iron ion content. F (5, 30) = 129.00, *P < 0.0001*. The data were analyzed using one‐way analysis of variance and all data are expressed as the mean ± standard deviation. **P < 0.05* represents a statistically significant difference between the two groups.

**S Figure 14. Tyr572Ala mutant of keap1 improved outcomes in Lcn2^fl/fl^Nestin^Cre^ mice with ICH.**

**A** Comparison of hemorrhagic areas in each group of mice. F_Interaction_(2, 24) = 69.22, *P < 0.0001*. **B** Longa scores in each group of mice. F_Interaction_(2, 54) = 14.54, *P < 0.0001*. **C** mNSS scores in each group of mice. F_Interaction_(2, 54) = 61.58, *P < 0.0001*. **D** The rotarod test measures the duration that mice can maintain their balance on a rotating rod. F_Interaction_(2, 54) = 99.77, *P < 0.0001*. **E** brain water content in each group of mice. F_Interaction_(2, 54) = 78.02, *P < 0.0001*. **F** Expression levels of GPX4 and xCT mRNA. GPX4: F_Interaction_(2, 12) = 202.80, *P < 0.0001*. xCT: F_Interaction_(2, 12) = 37.89, *P < 0.0001*. **G** Expression levels of GPX4 and xCT protein. **H** Quantification of results in Panel G. GPX4: F_Interaction_(2, 12) = 93.00, *P < 0.0001*. xCT: F_Interaction_(2, 12) = 105.30, *P < 0.0001*. **I** Assess the level of apoptosis in the brain tissue surrounding the hemorrhage in each group of mice using flow cytometry. F_Interaction_(2, 12) = 63.07, *P < 0.0001*. **J** Iron ion staining.  **K** Detection of iron ion content in brain tissue surrounding hemorrhage. F_Interaction_(2, 24) = 79.72, *P < 0.0001*. **L** Expression levels of IL-1β in the surrounding brain tissue of mice in each group. F_Interaction_(2, 30) = 34.97, *P < 0.0001*. **M** Expression levels of IL-1β in the serum of each group of mice. F_Interaction_(2, 30) = 68.45, *P < 0.0001*. **N** Expression levels of IL-4 in the surrounding brain tissue of mice in each group. F_Interaction_(2, 30) = 20.52, *P < 0.0001*. **O** Expression levels of IL-4 in the serum of each group of mice. F_Interaction_(2, 30) = 136.10, *P < 0.0001*. The data were analyzed using two‐way analysis of variance and all data are expressed as the mean ± standard deviation. **P < 0.05* represents a statistically significant difference between the two groups. ns: no statistical difference.

S Figure 15. Expression levels of TNF-α, IL-18 and IL-10 in the surrounding brain tissue and serum of each group of mice.

**A** Expression levels of TNF-α, IL-18 and IL-10 in the surrounding brain tissue of mice in each group. TNF-α: F_Interaction_(2, 30) = 23.37, *P < 0.0001*. IL-18: F_Interaction_(2, 30) = 22.20, *P < 0.0001*. IL-10: F_Interaction_(2, 30) = 50.49, *P < 0.0001*. **B** Expression levels of TNF-α, IL-18 and IL-10 in the serum of each group of mice. TNF-α: F_Interaction_(2, 30) = 54.31, *P < 0.0001*. IL-18: F_Interaction_(2, 30) = 16.21, *P < 0.0001*. IL-10: F_Interaction_(2, 30) = 44.78, *P < 0.0001*. The data were analyzed using two‐way analysis of variance and all data are expressed as the mean ± standard deviation. **P < 0.05* represents a statistically significant difference between the two groups. ns: no statistical difference.
